# Benchmarking break-junction techniques: electric and thermoelectric characterization of naphthalenophanes†

**DOI:** 10.1039/d4nr00704b

**Published:** 2024-04-15

**Authors:** Juan Hurtado-Gallego, Sebastiaan van der Poel, Matthias Blaschke, Almudena Gallego, Chunwei Hsu, Rubén López-Nebreda, Marcel Mayor, Fabian Pauly, Nicolás Agraït, Herre S. J. van der Zant

**Affiliations:** a Departamento de Física de la Materia Condensada, Universidad Autónoma de Madrid 28049 Madrid Spain nicolas.agrait@uam.es; b Kavli Institute of Nanoscience, Delft University of Technology Lorentzweg 1 2628 CJ Delft The Netherlands H.S.J.vanderZant@tudelft.nl; c Institute of Physics and Center for Advanced Analytics and Predictive Sciences, University of Augsburg 86159 Augsburg Germany fabian.pauly@uni-a.de; d Department of Chemistry, University of Basel St. Johanns-Ring 19 4056 Basel Switzerland marcel.mayor@unibas.ch; e Institute for Nanotechnology, Karlsruhe Institute of Technology (KIT) P. O. Box 3640 76021 Karlsruhe Germany; f Lehn Institute of Functional Materials, School of Chemistry, Sun Yat-Sen University Guangzhou 510274 P. R. China; g Condensed Matter Physics Center (IFIMAC) and Instituto Universitario de Ciencia de Materiales ‘Nicolás Cabrera’ (INC), Universidad Autónoma de Madrid 28049 Madrid Spain

## Abstract

Break-junction techniques provide the possibility to study electric and thermoelectric properties of single-molecule junctions in great detail. These techniques rely on the same principle of controllably breaking metallic contacts in order to create single-molecule junctions, whilst keeping track of the junction's conductance. Here, we compare results from mechanically controllable break junction (MCBJ) and scanning tunneling microscope (STM) methods, while characterizing conductance properties of the same novel mechanosensitive *para*- and *meta*-connected naphtalenophane compounds. In addition, thermopower measurements are carried out for both compounds using the STM break junction (STM-BJ) technique. For the conductance experiments, the same data processing using a clustering analysis is performed. We obtain to a large extent similar results for both methods, although values of conductance and stretching lengths for the STM-BJ technique are slightly larger in comparison with the MCBJ. STM-BJ thermopower experiments show similar Seebeck coefficients for both compounds. An increase in the Seebeck coefficient is revealed, whilst the conductance decreases, after which it saturates at around 10 μV K^−1^. This phenomenon is studied theoretically using a tight binding model. It shows that changes of molecule-electrode electronic couplings combined with shifts of the resonance energies explain the correlated behavior of conductance and Seebeck coefficient.

## Abstract 2

Break-junction techniques provide the possibility to study electric and thermoelectric properties of single-molecule junctions in great detail.

## Graphical abstract

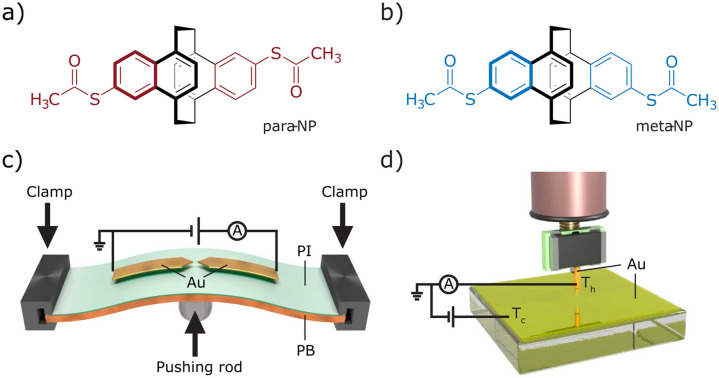

## Introduction

The vision of molecular electronics inspired numerous experimental and theoretical studies in the last decades.^1,2^ A main theme of the field is to explore both the potential and the limitation of single molecules as functional units of electronic circuits such as diodes, switches or thermoelectric nanodevices.^3–6^ The in-depth analysis of electric and thermoelectric properties of single-molecule junctions is essential for scientific advances.^7^ Different experimental approaches have been used for the study of single-molecule junctions, with the most popular one being the break-junction (BJ) technique, comprising mechanically controlled break junction (MCBJ) and scanning tunneling microscopy break junction (STM-BJ) methods. Both approaches have in common that the electrode distance can be varied with sub-Ångström resolution during transport experiments, making them particularly appealing for the analysis of mechanosensitive systems. We have investigated the mechanosensitivity of cyclophanes of various dimensions, ranging from compact [2.2]paracyclophanes^8–10^ to considerably larger porphyrin cyclophanes,^11–13^ which both displayed a complex interplay between mechanical stimulation and electric transport properties.

**Fig. 1:**
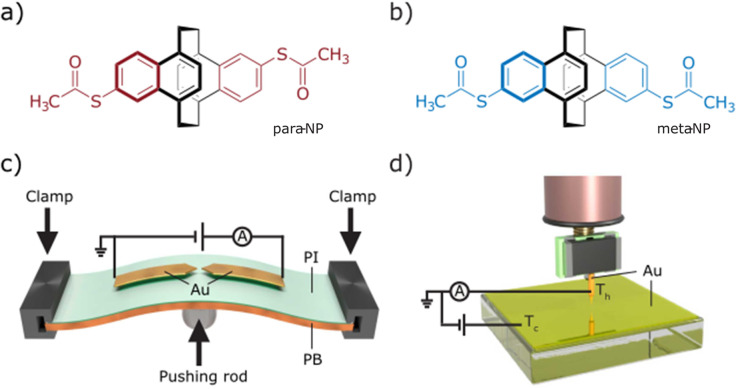
(a and b) Schemes of *para*-NP and *meta*-NP molecules. (c and d) Schemes of the MCBJ and STM-BJ setups, showing the arrangement for the electric characterization. Additionally, in the STM a temperature gradient between the Au tip and the Au substrate is established to enable thermopower measurements.

Here, we report the optimization of compact naphthalenophane model compounds *para*-NP (Fig. 1a) and *meta*-NP (Fig. 1b), together with their electric and thermoelectric transport characterization in terms of the two mentioned break-junction techniques: MCBJ^14^ (Fig. 1c) and STM-BJ^15^ (Fig. 1d). Using these two techniques yields insights into the interdependent mechanical and electronic behavior of the molecular junctions. The two naphtalenophanes differ in the substitution pattern of the thiol anchor groups and thus enable to investigate correlations between transport and substitution pattern, as well as differences in the operation modes of both experimental set-ups. To compare the measurement methods, the same data analysis technique was applied to the collected data sets. Additionally, Seebeck coefficient measurements using the STM-BJ technique allow us to study not only the electronic transport but also the thermoelectric transport properties of the compounds. In this report, we focus on the electrical and thermoelectrical properties of the compounds, leaving further details and in particular the mechanosensitive properties of these molecules for a subsequent work.

## Experimental

### Synthesis

Mechanosensitive [2.2]paracyclophanes (PCPs) that have been investigated so far were decorated with phenyl substituents exposing the anchor group.^8,9^ Either the torsion angle between both aromatic systems^9^ or the sp-hybridized carbon atoms of the ethynyl linker^8^ obstructed the electronic transparency of the structure. With the intention to maximize the electronic coupling of the anchor-group bearing phenyl ring and the mechanosensitive PCP subunit, the naphthalenophane model compounds were designed.

**Scheme 1:**
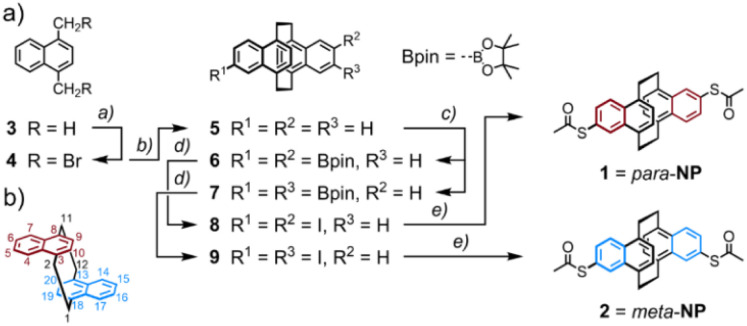
(a) Synthesis of the *anti*-[2.2](1,4)naphthalenophanes *para*-NP (1) and *meta*-NP (2), exposing a pair of acetyl protected thiol anchor groups. Reagents and conditions: (a) 3 eq. NBS, 10 mol% (C_6_H_5_CO)_2_O_2_, CH_2_Cl_2_, 55 °C, 18 h, 78%; (b) 2.5 eq. SmI_2_, THF, r.t., 4 h, 89%; (c) (1.) 10 mol% [Ir(COD)(OMe)]_2_, 20 mol% 4,4′-di-*t*Bu-bipy, 2.5 eq. bis(pinacolato)diboron, THF, reflux, 18 h, 77%, as mixture of regioisomers 6 and 7; (2.) separation of regioisomers by HPLC, isolated yields: 29% of 6 and 31% of 7; (d) (1.) 10 eq. CH_3_B(OH)_2_, CH_2_Cl_2_, CF_3_COOH, r.t., 2 d, evaporation to dryness; (2.) 1 eq. I_2_, K_2_CO_3_, CH_3_CN, reflux, 4 h; isolated yields: 65% of 8 and 77% of 9; (e) 4 eq. CH_3_COSK, 2 mol% Pd_2_dba_3_, 4 mol% xantphos, CH_3_C_6_H_5_/CH_3_COCH_3_: 2/1, seal tube, 70 °C, 2 h; isolated yields: 69% of 1 and 84% of 2. (b) Positional numbering in the *anti*-[2.2](1,4)naphthalenophane skeleton.

The syntheses of the model compounds labeled as *para*-NP (5,15-bis(acetylthio)-*anti*-[2.2](1,4)naphthalenophane (see Fig. 1a)) and *meta*-NP (5,16-bis(acetylthio)-*anti*-[2.2](1,4)naphthalenophane (see Fig. 1b)) are summarized in Scheme 1a, and the corresponding synthetic protocols and characterization data are provided in the ESI.† The target structures *para*-NP (1) and *meta*-NP (2) were obtained by functionalizing the parent structure *anti*-[2.2](1,4)naphthalenophane (5). Note, the numbering of the positions in the parent structure 5, which is depicted in Scheme 1b. For simplicity we refer to the target structures with trivial names, pointing at the arrangement of the anchor groups in the *anti*-[2.2](1,4)naphthalenophane core by using *para*-NP for 1 and *meta*-NP for 2.

The parent *anti*-[2.2](1,4)naphthalenophane skeleton was synthesized with a slight modification of a reported protocol.^16^ Treating commercially available 1,4-dimethylnaphthalene 3 with *N*-bromosuccinimide (NBS) provided 1,4-bis(bromomethyl)naphthalene 4 in good isolated yields.^17^ Exposing 4 to samarium(ii) diiodide in THF gave the parent *anti*-[2.2](1,4)naphthalenophane 5 in excellent 89% isolated yield. A C–H activation-based protocol for the borylation of naphthalenes,^18^ applied to 5, provided the regioisomers 6 and 7, which were isolated as mixture in 77% yield. In several HPLC runs the mixture was separated into the di-borylated regioisomers 6 and 7, which were isolated in 29% and 31% yield, respectively. Hydrolysis of the boronic ester followed by substitution of the boronic acids by iodine gave the regioisomers 8 and 9 in 65% and 77% isolated yields, respectively. With a palladium catalyzed protocol,^19^ the iodines of 8 and 9 were substituted by acetylsulfanyl groups, giving the target structures 1 (*para*-NP) and 2 (*meta*-NP) in 69% and 84% isolated yields, respectively.

**Fig. 2:**
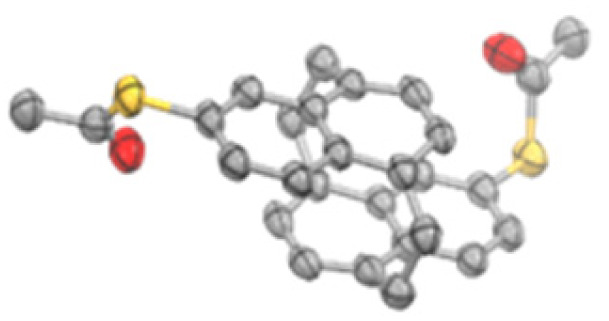
Solid-state structure of 5,16-bis(acetylthio)-*anti*-[2.2](1,4)naphthalenophane 2 (*meta*-NP). Oak Ridge Thermal Ellipsoids (ORTEPs) are plotted on a 50% probability level. Hydrogen atoms and solvent molecules are omitted for clarity. Crystallographic data are deposited under CCDC 2267417.†

The new compounds were fully characterized by ^1^H- and ^13^C-NMR spectroscopy and mass spectrometry. The identities of both target structures were corroborated by their analytical data. In addition, single crystals suitable for X-ray analysis were obtained by slow evaporation of the solvent from a solution of 2 (*meta*-NP) in CHCl_3_. The solid-state structure, displayed in Fig. 2, confirms the assigned substitution pattern and the identity of 2.

### Conductance measurements

Single-molecule studies were performed by employing break-junction techniques with two different home-built set-ups: a MCBJ and a STM-BJ, both operating at room temperature under ambient conditions.

The MCBJ technique entails the repeated process of breaking and making a thin, initially lithographically defined, gold (Au) nanowire on a layer of polyimide (PI) on top of a flexible phosphorous bronze (PB) substrate (see Fig. 1c). Using a three-point bending mechanism containing two clamps, in which the substrate is placed, and a pushing rod connected to a piezoelectrical element, the gold nanowires can be broken and made repeatedly. Upon breaking the nanowire, two atomically sharp electrodes are formed, and the distance in between them can be manipulated with picometer precision. While bending and ultimately breaking the gold nanowire, we apply a bias voltage (*V*_bias_ = 100 mV) across the structure and continuously monitor the current as a function of the electrode separation distance.^20^ Molecules are deposited by dropcasting a dicholoromethane (DCM) solution of them directly onto the MCBJ sample. The concentration is 5 μM for both molecules. With molecules deposited onto the sample, the breaking of the gold wire results in either the formation of empty (tunneling) junctions or molecular junctions. It is noteworthy that on the gold surface, probably in the presence of traces of water, the acetyl protection groups cleave and covalent sulfur–gold bonds are formed.^21^ The formation of these rather robust bonds, mounting the molecule at the interface, is of particular importance when mechanical properties of the molecule in the junction are under investigation. After attaining a junction, the electrodes are separated until the current drops below the detection level of the measurement system, *i.e.*, the noise floor. Afterwards, the electrodes are brought into contact again, until a current is measured corresponding to a multiple of *G*_0_ in conductance, whereafter the breaking process is repeated. Here, *G*_0_ = 2*e*^2^/*h* is the quantum of conductance, where *e* is the electron charge and *h* is Planck's constant.

The STM-BJ method creates single-molecule junctions using a homemade STM. The employed STM is a system capable of measuring the tunneling current through a tip and a conductive substrate, both acting as electrodes. Mechanically cut Au wires (0.25 mm diameter, 99.99% purity, Goodfellow) are used as tips. The tip is connected by a tip holder to a piezoelectric tube, which moves the tip vertically and horizontally with a resolution of 10–20 pm. The tip is placed above an Au (111) substrate (11 × 11 mm^2^, Arrandee; Fig. 1d), where molecules have been deposited by immersion of the pre-annealed substrates for 20 minutes in a 1 mM DCM solution of the respective compound. Subsequent blowing with nitrogen gas eliminates the remaining solvent from the substrate. The tip is approached to the sample until the conductance of the junction is several times *G*_0_; hereafter, it is retracted until the current drops below the noise floor of the system. During the retraction process one or several molecules, deposited on the sample, may be trapped between the tip and the sample, forming a molecular junction. The current is recorded during the entire piezo movement, and the breaking process is repeated many times.^15^

**Fig. 3:**
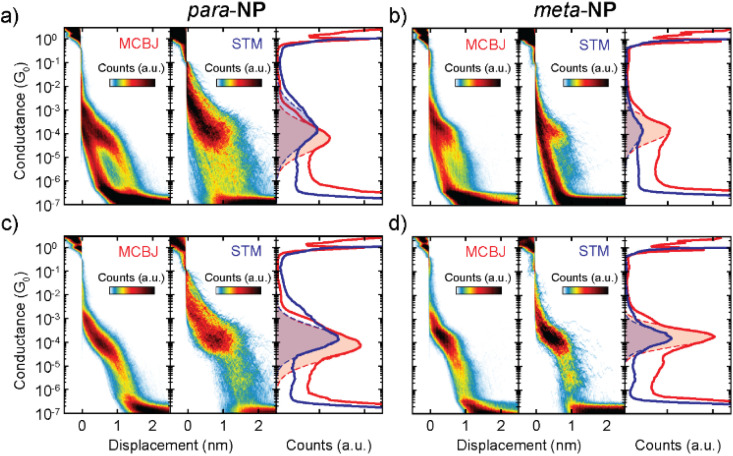
(a) Conductance (*G*) measurements of the *para*-NP molecule. Left and central panels display the two-dimensional conductance *vs.* displacement histograms of all the measured traces, obtained with the MCBJ and STM-BJ techniques. They consist of 10 000 and 2200 traces, respectively. The right panel shows the corresponding one-dimensional conductance histograms of MCBJ (red) and STM (blue) measurements. Dashed lines are Gaussian fits of the respective peaks for STM and MCBJ. (b) Same as (a) but for the *meta*-NP molecule. (c) and (d) Same as (a) and (b), but now only including the molecular traces for the *para*-NP and *meta*-NP molecules, respectively. These traces were found by performing clustering with two classes.

Fig. 3 shows conductance (*G*) measurements of the *para*-NP (a and c) and *meta*-NP (b and d) compounds, using both break-junction methods. All measured traces for both compounds are included in Fig. 3a and b, located in the left (MCBJ) and middle (STM) panels of the two-dimensional conductance *vs.* displacement histograms. The right panels show the corresponding one-dimensional conductance histograms. The red (MCBJ) and blue (STM) drawn lines represent the main conductance peak with the respective Gaussian distribution fits (dashed lines and colored areas). Differences in the noise saturation level of both compounds emerge from the current amplifiers used for the two measurement techniques, *i.e.*, a logarithmic amplifier for the MCBJ method and a linear one for the STM method. The histograms in Fig. 3c and d are compiled from conductance–distance traces corresponding to molecular junctions of both compounds and methods (with molecular yields between 22–41%), which are selected from the total number of curves using a non-supervised clustering technique based on Matlab's ‘*k*-means’ function.^22,23^ Most probable conductance values (*G*_m_) and apparent stretching. Lengths (*L*_s_) are obtained for each compound and method. They are summarized in Table 1 (see the ESI† for further details).

**Table 1:** Most probable conductance (*G*_m_) and apparent stretching length (*L*_s_) of all molecular traces for all compounds, as extracted from Fig. 3c and d, comparing MCBJ and STM-BJ techniques. Uncertainties, obtained from the standard deviations of the Gaussian fits, are specified for each value. Additionally, thermopower values are shown, as measured with the STM-BJ method. Uncertainties, obtained from the standard error of the linear regression, are indicated for each value

*para*-NP				*meta*-NP			
MCBJ		STM-BJ		MCBJ		STM-BJ	
*G*_m_ (*G*_0_)	*L*_s_ (nm)	*G*_m_ (*G*_0_)	*L*_s_ (nm)	*G*_m_ (*G*_0_)	*L*_s_ (nm)	*G*_m_ (*G*_0_)	*L*_s_ (nm)
(7.9 ± 4.8) × 10^−5^	0.7 ± 0.1	(1.5 ± 0.9) × 10^−4^	1.2 ± 0.2	(1.7 ± 0.7) × 10^−4^	0.6 ± 0.1	(1.6 ± 0.9) × 10^−4^	0.7 ± 0.1
—		*S* (μV K^−1^)		—		*S* (μV K^−1^)	
		10.1 ± 0.2				10.0 ± 0.2	

Similar *G*_m_ and *L*_s_ values are obtained for the *meta*-NP compound using the MCBJ and STM-BJ methods, yielding 1.7 × 10^−4^*G*_0_ and 1.6 × 10^−4^*G*_0_ for the conductance and 0.6 nm and 0.7 nm for the stretching lengths, respectively. This indicates good agreement between the two different measurement techniques. For the *para*-NP compound *G*_m_ and *L*_s_ values however differ for both measurement methods, yielding mean conductance values of 1.5 × 10^−4^*G*_0_ and 7.9 × 10^−5^*G*_0_, and *L*_s_ values of 0.7 nm and 1.2 nm for the MCBJ and STM, respectively. Considering the standard deviations, the conductances of both methods are still compatible with each other. The variations in conductance and stretching length may be due to the different shapes of the electrodes which could lead to varied contact geometries or modified local molecular concentrations in the junctions. Additional MCBJ measurements on *para*-NP with a higher molecular concentration of 50 μM, presented in the ESI (Fig. S2†), indicate however that changes in the concentration do not play a significant role. The fact that the breaking of the molecular junctions for the MCBJ appears smoother, as can be seen in the individual breaking traces shown in Fig. S4 of the ESI,† suggests that differences in conductance and stretching length obtained in the two setups may also be a consequence of the higher mechanical stability of the MCBJ. Regarding the conductance uncertainties, shown in Table 1, the higher mechanical stability of the MCBJ is indeed reflected in smaller errors for all the high *G* values.

Applying the same clustering technique to the molecular traces, additional lower conductance plateaus are found in the two-dimensional histograms for all compounds using both experimental methods (see the ESI† for further details). These lower conductance plateaus could indicate the existence of other stable configurations of the molecule inside the junction. Further studies on the origin of the multiple conductance values are required. The current hypothesis is that they arise from different configurations between the molecule and the electrode, which might or might not involve the anchor groups.^24^

### Seebeck coefficient measurements

Seebeck coefficient or equivalently thermopower measurements were performed with a temperature difference, Δ*T*, established between both electrodes using the STM-BJ setup. We heated the tip using a 1 kΩ surface resistor, placed on top of the tip holder, while the substrate remained at room temperature. Voltage ramps of ±10 mV were applied during the formation of molecular junctions, and the resulting current–voltage curves were recorded (as detailed in section S8 of the ESI†). This procedure allows to measure simultaneously the conductance and the thermovoltage, *V*_th_, at different electrode displacements. Here, *V*_th_ is defined as the voltage response between the sample and the tip of the STM.

**Fig. 4:**
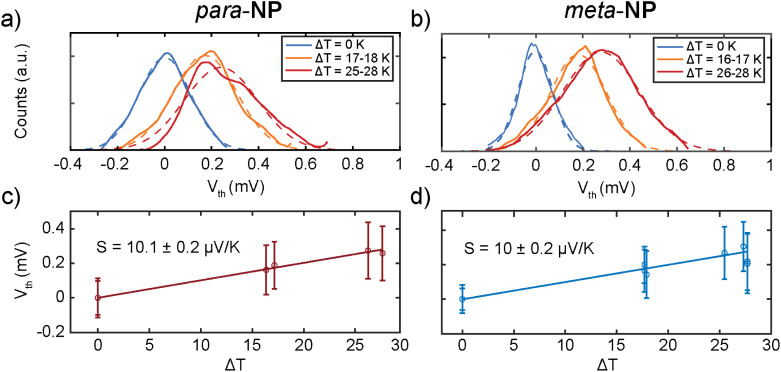
(a and b) One-dimensional histograms of all thermovoltage (*V*_th_) points, measured in three different temperature ranges (Δ*T*) for the (a) *para*-NP and (b) *meta*-NP compounds, respectively. (c and d) Mean Seebeck coefficient *S* values of (c) *para*-NP and (d) *meta*-NP and their respective uncertainties, obtained from the standard error of each linear regression. The thermopower *S* is obtained from the linear regression (solid drawn line) of all *V*_th_*vs.* Δ*T* points. Mean values and standard deviations of each *V*_th_ set of measurements are displayed as empty circles and error bars, respectively.

Additionally, *V*_th_ was measured with different Δ*T* between 0 and 28 K, as shown in Fig. 4a and b for the *para*-NP and *meta*-NP compounds, respectively. Gaussian distributions were fitted to the different ensembles of thermovoltage, yielding for each of them the mean thermovoltage and the standard deviation. As a function of the temperature difference between tip and sample, these are plotted in Fig. 4c and d as empty circles and error bars, respectively. From the thermovoltage of the system (*V*_th_ = (*S* − *S*_lead_)Δ*T*) we extract the Seebeck coefficient of the junction (*S*), taking into account the thermoelectrical influence of the copper lead (*S*_lead_)^25^ that connects the tip to the rest of the experiment.

The Seebeck coefficient was obtained from the slope of linear fits to the *V*_th_*vs.* Δ*T* data points, and the values are displayed in Fig. 4c and d and Table 1. Positive *S* values indicate hole transport mainly through the HOMO, as reported before for thiol anchoring groups.^26,27^ Similar *S* values of around 10 μV K^−1^ are obtained for both compounds, showing negligible influence of the substitution patterns on the thermoelectric response.

**Fig. 5:**
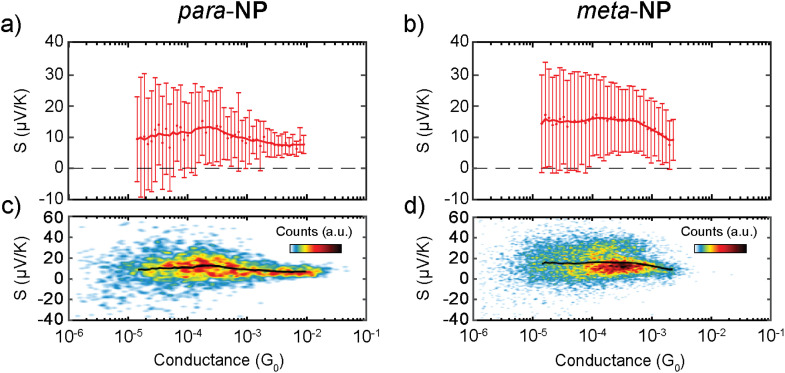
(a and b) Dots and error bars represent mean Seebeck coefficient values and standard deviations of (a) *para*-NP and (b) *meta*-NP as a function of conductance. Thick red solid lines show gently smoothed mean Seebeck coefficient values. (c and d) Two-dimensional Seebeck coefficient *vs.* conductance histograms, containing all measured data points for (c) *para*-NP and (d) *meta*-NP. The black solid line in each panel represents the mean Seebeck coefficient along the conductance. It is identical to the thick red solid line in panel (a) or (b), respectively.

Mean *S vs. G* traces of each compound are displayed in Fig. 5a and b with their respective standard deviations, and two-dimensional *S vs. G* histograms of both compounds are shown in Fig. 5c and d, where all the measured Seebeck coefficient points are included. An increase in *S* as the conductance decreases in the range of 10^−2^–10^−4^*G*_0_ is followed by a saturation at low *G* values for both molecules, as shown in Fig. 5a and b. This behavior is also observed in individual *S vs.* displacement traces. Examples of conductance *vs.* displacement traces with *S* measurements along the molecular plateau are shown in Fig. S7,† exhibiting an increase in *S* followed by a saturation for lower *G* and larger electrode displacement distances.

## Theoretical

According to Table 1, the overall behavior of *para*-NP and *meta*-NP in terms of conductance and thermopower is similar. In this work we do not aim at explaining individual conductance-distance traces or the mechanosensitive response, and an atomistic modeling of the molecular junctions will be presented in a subsequent manuscript. Instead, we study here a generic model that can be applied to both molecules and try, in particular, to understand the general trend of an increasing thermopower for a decreasing conductance, as visible in Fig. 5.

**Fig. 6:**
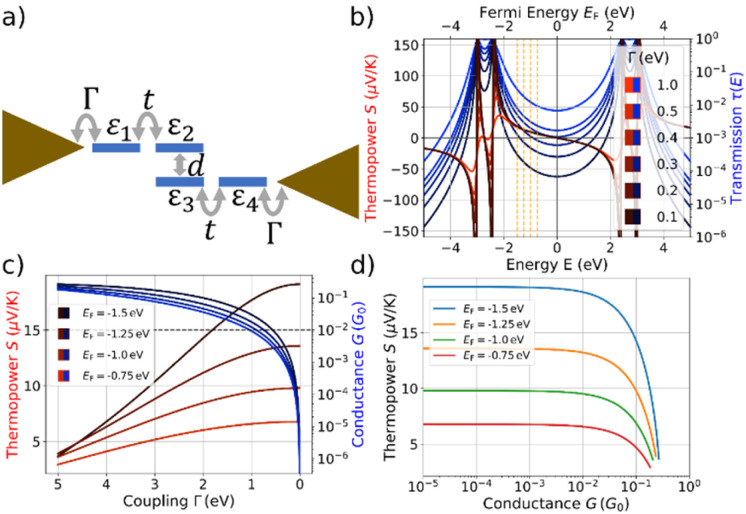
(a) Four-site tight-binding model with on-site energies *ε*_*i*_ and *i* = 1, …4, intradeck hopping terms *t* and interdeck hopping *d*, and a symmetric coupling *Γ* to left and right electrodes. Model results are obtained for *ε*_*i*_ = 0 for all *i*, *t* = 2.7 eV and *d* = 0.6 eV. For evaluations of the conductance *G* and thermopower *S* we assume a temperature of *T* = 300 K. (b) The thermopower *S* is shown in shades of red and the transmission *τ*(*E*) in shades of blue. The transmission is plotted as a function of energy (lower *x*-axis) for the indicated values of *Γ* and the thermopower as a function of the respective Fermi energy (upper *x*-axis) for the same values of *Γ*. (c) *S* and *G* plotted against the coupling strength *Γ*. The behavior is depicted for the four different Fermi energies, marked by vertical dashed orange lines in panel (b). The horizontal dashed line indicates a conductance of 10^−2^*G*_0_. (d) *S* plotted as a function of *G*, using the data from panel (c).

For this purpose we explore a four-site tight-binding model for the metal-molecule-metal junctions, where each phenyl ring is represented by a single site. The model is depicted in Fig. 6a. It contains on-site energies, *ε*_*i*_, for all four sites *i* = 1, …4, two distinct hopping parameters, *t* and *d*, which describe the couplings inside the naphthalene decks and between them, and a symmetric coupling to left and right electrodes, *Γ*. The parameters *t* and *d* are fitted using DFT calculations^28^ of the isolated molecule combined with the *G*_0_*W*_0_ correction.^29^ Further details can be found in the ESI.†

We model transport properties within the framework of the Landauer-Büttiker scattering theory^30^ using the wide-band limit approximation.^31^ From the energy-dependent electronic transmission we compute^32–34^ the conductance *G* = *G*_0_*K*_0_ and the thermopower *S* = −*K*_1_/(*eTK*_0_) with 
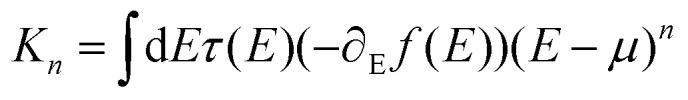
. Here, *f*(*E*) is the Fermi function, and *μ* the electrochemical potential, which we assume to be equal to the Fermi energy, *E*_F_. Additional information on the formalism can be found in the ESI.†

While stretching a molecular junction, the molecule, trapped between the macroscopic gold metallic electrodes, will adjust its position. During this process the variation of three distinct model parameters seems reasonable: (i) decreasing electronic couplings, *Γ*, between molecule and electrodes due to a reduced electronic overlap, (ii) variation of the interdeck hopping, *d*, due to geometric changes inside the molecule, and (iii) shifts of on-site energies, arising from varying charge transfer and screening between molecule and electrodes. We assume that changes in the intradeck hopping terms, *t*, between both benzene rings of the naphthalene units can be neglected due to strong covalent bonds within both naphthalene subunits.

We explore first, whether the characteristic coupled behavior of increasing *S* for reduced *G* can be explained by decreasing molecule-electrode electronic couplings, *Γ*.^35–39^ We assume that the electronic couplings to the electrodes are reduced in the stretching process towards the point of rupture, where the molecule is fully erected inside the junction. Fig. 6b displays the transmission and thermopower as a function of energy for different *Γ*. Note, that for the transmission we plot *τ*(*E*) as a function of *E* (lower *x*-axis) in Fig. 6b, while *S* is plotted *vs. E*_F_ due to the energy integration at finite *T* = 300 K (upper *x*-axis). As *Γ* decreases, broadenings of transmission resonances shrink, resulting in a reduced transmission or conductance at the four indicated Fermi energies inside the HOMO–LUMO gap (vertical dashed orange lines in Fig. 6b). The thermopower reaches its highest absolute values in the vicinity of molecular energy levels for low values of *Γ* due to the increased slope of *τ*(*E*) and decreased value of *τ*(*E*).

Thermopower values are shown in Fig. 6c at the marked Fermi energies together with the conductance, both plotted *vs.* a varying *Γ*. We find an increase of *S* from approximately 3.5 μV K^−1^ for all indicated Fermi energies to 6.8 μV K^−1^ (*E*_F_ = −0.75 eV), 9.8 μV K^−1^ (*E*_F_ = −1.0 eV) 13.6 μV K^−1^ (*E*_F_ = −1.25 eV) and 19.1 μV K^−1^ (*E*_F_ = −1.5 eV). Initially, the thermopower rises rather linearly with increasing *Γ* at the chosen values of *E*_F_, but saturates for low values of *Γ*, while the conductance simultaneously decays to low values.

The correlated behavior can best be perceived by plotting *S* as a function of *G*, as shown in Fig. 6d. This panel visualizes the saturated behavior of *S* in the off-resonant transport regime at low *G* and the decrease of *S* for increasing *G*, when *G* is plotted on a logarithmic scale.

A comparison of Fig. 6d with the experimental data in Fig. 5 shows a qualitative agreement of a growing *S* for decreasing *G*, followed by a subsequent saturation at low *G*. The experimental mean values for *para*-NP (*meta*-NP) start at approximately 5.90 μV K^−1^ for a conductance of 10^−2^*G*_0_ (4.53 μV K^−1^ for a conductance of 2 × 10^−3^*G*_0_) and saturate around 15.96 μV K^−1^ at a conductance of 3 × 10^−4^*G*_0_ (13.33 μV K^−1^ at a conductance of 3 × 10^−4^*G*_0_), which agrees favorably with the theoretically determined thermopower *S* especially for Fermi energies *E*_F_ = −1.25 eV and *E*_F_ = −1.5 eV. To achieve the variation in *S*, however, the range of conductance values *G* in the experimental data is around two orders of magnitude smaller than in the theoretical toy model. Consequently, we assume that the range of reasonable values for electrode-molecule couplings should be restricted to values *Γ* < 1.0 eV to stay in the experimentally observed window for the conductance, as indicated by the horizontal dashed line in Fig. 6c. For molecule–electrode couplings in the range 0 ≤ *Γ* ≤ 1.0 eV, unfortunately, the variation of *S* is significantly smaller than in the experimental data.

We analyze the effect of asymmetric molecule–electrode couplings, which might be realized during the stretching, in the ESI.† Here, a qualitatively similar behavior to the symmetric configuration is observed. The change in *Γ* (symmetric or asymmetric) qualitatively represents the measured data, yet, for a quantitative agreement, the variation of additional parameters appears to be important.

Similar to previous work on paracyclophanes,^8,9^ the interdeck coupling, *d*, between the naphthalene planes is expected to play a crucial role for quantum interference effects, leading to mechanosensitivity. Features from destructive quantum interference in conductance–distance traces occur, however, only at specific stretching distances during the stick-slip motion of the molecules on the gold electrodes.^8^ Variations in interdeck electronic coupling are thus not expected to be responsible for the global decrease of thermopower *S* with reduced conductance *G*, which happens on larger displacement distances than the stick-slip periods. Conductance–distance traces, as presented in the ESI (see especially Fig. S4†), indeed show multiple conductance oscillations, which we assign to destructive quantum interference effects, on a single conductance plateau. A detailed analysis of variations in *d* in our simple four-site model can be found in the ESI.† If we reduce the coupling, *d*, between the naphthalene planes (see Fig. 1a and b and Fig. 6a), the thermopower and conductance both decrease, which is in contradiction to the experimental data. Additionally, the relative change in *S* is rather small for the estimated parameter range. Thus, we conclude that a reduction of the interdeck hopping *d* is most likely not decisive for the experimentally observed global conductance-thermopower behavior in Fig. 5.

Another degree of freedom are the on-site energies, *ε*_*i*_, which can modify the energies, where transmission resonances occur. For sake of simplicity, we restrict ourselves to changes which uniformly shift all resonance energies to orthogonalize the effect to the preceding discussion. Within our modelling, uniform shifts of on-site energies can be translated to changes in the Fermi energy, allowing the effects to be estimated from Fig. 6c and d. We restrict values of the coupling strength to *Γ* < 1.0 eV to operate in the experimentally realized window of conductance values below 10^−2^*G*_0_. A quantitative agreement of theoretically computed thermopower and conductance with the experimental data is achieved, if the Fermi energy is gradually shifted from *E*_F_ = −0.75 eV to *E*_F_ = −1.5 eV, while *Γ* drops simultaneously. Since *E*_F_ = 0 means that the Fermi energy is located in the middle of the HOMO–LUMO gap, this corresponds to a decreasing separation of the HOMO level from the electrode Fermi energy during the stretching. To obtain the saturation in the thermopower *S* for low conductance values, the shift of the molecular orbital energies must also saturate during the stretching. Although the precise mechanism cannot be elucidated due to the simplicity of our model, the thermopower is very sensitive to the alignment of molecular energy levels to the electrodes’ Fermi energy. Such molecular energy level shifts and their influence on the thermopower have been reported in the literature for various molecules and models.^35–37,40,41^

Overall, the four-site tight-binding model offers a framework for elucidating the significant mechanisms, leading to the global trends of *S* and *G* in the experimental results. Based on our analysis, a reduction in *Γ* during the stretching combined with shifts in orbital energies that bring the HOMO level closer to the Fermi energy are responsible for the observed behavior.

## Conclusions

*para*- and *meta*-connected naphthalenophane compounds were electrically characterized using two different break-junction techniques, *i.e.*, MCBJ and STM-BJ. The molecules were designed to be more rigid and to have higher electrical conductance than previously measured paracyclophane compounds.^8,9^ Conductance measurements with both techniques show a good agreement for the *para*- and *meta*-connected naphthalenophanes, which exhibit strong similarities in their electrical conductance. The higher mechanical stability of the MCBJ leads to smaller conductance uncertainties for the two compounds. The usage of different current amplifiers is not directly reflected in the conductance values of both molecules but just in the noise saturation level of the measurements. As anticipated, the conductance is higher than that of the previously studied paracyclophane derivatives.^8,9^ MCBJ has the advantage of a better mechanical stability for the conductance measurements. On the other hand, the higher versatility of the STM makes it easier to implement thermopower measurements. We find similar thermopower values for *para*- and *meta*-connected naphthalenophanes of around 10 μV K^−1^ and a global increase of the thermopower up to around 15 μV K^−1^ as the conductance decreases to values down to 10^−4^*G*_0_. A tight-binding model suggests that this is related to a decrease of the electronic couplings of the molecule to the electrodes combined with shifts of the molecular orbital energies to become more resonant.

## Author Contributions

J. H.-G., R. L.-N., and N. A. contributed to the STM experimental part. S. v. d. P., C. H., and H. S. J. v. d. Z. contributed to the MCBJ experimental measurements. M. B. and F. P. performed the theoretical modelling. A. G. and M. M. synthesized the compounds.

## Conflicts of interest

There are no conflicts to declare.

## Supplementary Material

NR-016-D4NR00704B-s001

NR-016-D4NR00704B-s002

## References

[cit1] Ratner M. (2013). Nat. Nanotechnol..

[cit2] Heath J. R. (2009). Annu. Rev. Mater. Res..

[cit3] Harzheim A. (2018). Mater. Sci. Technol..

[cit4] Zhang J. L., Zhong J. Q., Lin J. D., Hu W. P., Wu K., Xu G. Q., Wee A. T. S., Chen W. (2015). Chem. Soc. Rev..

[cit5] Capozzi B., Xia J., Adak O., Dell E. J., Liu Z. F., Taylor J. C., Neaton J. B., Campos L. M., Venkataraman L. (2015). Nat. Nanotechnol..

[cit6] Perrin M. L., Galán E., Eelkema R., Thijssen J. M., Grozema F., van der Zant H. S. J. (2016). Nanoscale.

[cit7] Rincón-García L., Evangeli C., Rubio-Bollinger G., Agraït N. (2016). Chem. Soc. Rev..

[cit8] Stefani D., Weiland K. J., Skripnik M., Hsu C., Perrin M. L., Mayor M., Pauly F., van der Zant H. S. J. (2018). Nano Lett..

[cit9] Reznikova K., Hsu C., Schosser W. M., Gallego A., Beltako K., Pauly F., van der Zant H. S. J., Mayor M. (2021). J. Am. Chem. Soc..

[cit10] Blaschke M., Pauly F. (2023). J. Chem. Phys..

[cit11] Zwick P., Hsu C., El Abbassi M., Fuhr O., Fenske D., Dulić D., van der Zant H. S. J., Mayor M. (2020). J. Org. Chem..

[cit12] Schosser W. M., Hsu C., Zwick P., Beltako K., Dulić D., Mayor M., van der Zant H. S. J., Pauly F. (2022). Nanoscale.

[cit13] Hsu C., Schosser W. M., Zwick P., Dulić D., Mayor M., Pauly F., van der Zant H. S. J. (2022). Chem. Sci..

[cit14] Reed M. A., Zhou C., Muller C. J., Burgin T. P., Tour J. M. (1997). Science.

[cit15] Evangeli C., Gillemot K., Leary E., González M. T., Rubio-Bollinger G., Lambert C. J., Agraït N. (2013). Nano Lett..

[cit16] Takahashi S., Mori N. (1991). J. Chem. Soc., Perkin Trans. 1.

[cit17] Cangelosi V. M., Sather A. C., Zakharov L. N., Berryman O. B., Johnson D. W. (2007). Inorg. Chem..

[cit18] Yamamoto T., Ishibashi A., Koyanagi M., Ihara H., Eichenauer N., Suginome M. (2017). Bull. Chem. Soc. Jpn..

[cit19] Kim M., Yu S., Kim J. G., Lee S. (2018). Org. Chem. Front..

[cit20] Martin C. A., Smit R. H. M., van Egmond R., van der Zant H. S. J., van Ruitenbeek J. M. (2011). Rev. Sci. Instrum..

[cit21] Leary E., La Rosa A., González M. T., Rubio-Bollinger G., Agraït N., Martín N. (2015). Chem. Soc. Rev..

[cit22] Cabosart D., El Abbassi M., Stefani D., Frisenda R., Calame M., van der Zant H. S. J., Perrin M. L. (2019). Appl. Phys. Lett..

[cit23] Zotti L. A., Bednarz B., Hurtado-Gallego J., Cabosart D., Rubio-Bollinger G., Agraït N., van der Zant H. S. J. (2019). Biomolecules.

[cit24] Schneebeli S. T., Kamenetska M., Cheng Z., Skouta R., Friesner R. A., Venkataraman L., Breslow R. (2011). J. Am. Chem. Soc..

[cit25] Cusack N., Kendall P. (1956). Proc. Phys. Soc..

[cit26] Rincón-García L., Evangeli C., Rubio-Bollinger G., Agraït N. (2016). Chem. Soc. Rev..

[cit27] Ismael A., Wang X., Bennett T. L. R., Wilkinson L. A., Robinson B. J., Long N. J., Cohen L. F., Lambert C. J. (2020). Chem. Sci..

[cit28] Franzke Y. J., Holzer C., Andersen J. H., Begušić T., Bruder F., Coriani S., Della Sala F., Fabiano E., Fedotov D. A., Fürst S., Gillhuber S., Grotjahn R., Kaupp M., Kehry M., Krstić M., Mack F., Majumdar S., Nguyen B. D., Parker S. M., Pauly F., Pausch A., Perlt E., Phun G. S., Rajabi A., Rappoport D., Samal B., Schrader T., Sharma M., Tapavicza E., Treß R. S., Voora V., Wodyński A., Yu J. M., Zerulla B., Furche F., Hättig C., Sierka M., Tew D. P., Weigend F. (2023). J. Chem. Theory Comput..

[cit29] van Setten M. J., Weigend F., Evers F. (2013). J. Chem. Theory Comput..

[cit30] Cuevas J. C. and Scheer E., Molecular Electronics: An Introduction to Theory and Experiment, World Scientific Series in Nanoscience and Nanotechnology, 2nd edn, 2017

[cit31] Verzijl C. J. O., Seldenthuis J. S., Thijssen J. M. (2013). J. Chem. Phys..

[cit32] Sivan U., Imry Y. (1986). Phys. Rev. B: Condens. Matter Mater. Phys..

[cit33] Esfarjani K., Zebarjadi M., Kawazoe Y. (2006). Phys. Rev. B: Condens. Matter Mater. Phys..

[cit34] Müller K. H. (2008). J. Chem. Phys..

[cit35] Park S., Jang J., Yoon H. J. (2021). J. Phys. Chem. C.

[cit36] Torres A., Pontes R. B., da Silva A. J. R., Fazzio A. (2015). Phys. Chem. Chem. Phys..

[cit37] Fujii S., Montes E., Cho H., Yue Y., Koike M., Nishino T., Vázquez H., Kiguchi M. (2022). Adv. Electron. Mater..

[cit38] Kobayashi S., Kaneko S., Fujii S., Nishino T., Tsukagoshi K., Kiguchi M. (2019). Phys. Chem. Chem. Phys..

[cit39] Vacek J., Chocholoušová J. V., Stará I. G., Starý I., Dubi Y. (2015). Nanoscale.

[cit40] Popp M. A., Erpenbeck A., Weber H. B. (2021). Sci. Rep..

[cit41] Bruot C., Hihath J., Tao N. (2012). Nat. Nanotechnol..

