# Supplementary material for: Benchmarking break-junction techniques: electric and thermoelectric characterization of naphthalenophanes
Source: Nanoscale. 2024 Apr 15;16(22):10751–9. doi: 10.1039/d4nr00704b (PMC11154865; doi:10.1039/d4nr00704b)
Supplement: NR-016-D4NR00704B-s001 [file NR-016-D4NR00704B-s001.pdf]

Electronic Supplementary Material (ESI) for Nanoscale.
This journal is © The Royal Society of Chemistry 2024

Supporting Information

Benchmarking break-junction techniques: Electric and thermoelectric characterization of naphthalenophanes

Juan Hurtado-Gallego [a, ‡], Sebastiaan van der Poel [b, ‡], Matthias Blaschke [c, ‡], Almudena Gallego [d], Chunwei Hsu [b], Rubén López-Nebreda [a], Marcel Mayor [d, e, f,], Fabian Pauly [c,*], Nicolás Agraït [a, g,*], Herre S. J. van der Zant [b,*]*

[a]*Departamento de Física de la Materia Condensada, Universidad Autónoma de Madrid, 28049 Madrid , Spain*

[b]*Kavli Institute of Nanoscience, Delft University of Technology, Lorentzweg 1, 2628 CJ Delft, The Netherlands*

[c]*Institute of Physics and Center for Advanced Analytics and Predictive Sciences, University of Augsburg, 86159 Augsburg, Germany*

[d]*Department of Chemistry, University of Basel, St. Johanns-Ring 19, 4056 Basel, Switzerland*

[e]*Institute for Nanotechnology, Karlsruhe Institute of Technology (KIT), P. O. Box 3640, 76021 Karlsruhe, Germany*

[f]*Lehn Institute of Functional Materials, School of Chemistry, Sun Yat-Sen University, Guangzhou 510274, P. R. China*

[g]*Condensed Matter Physics Center (IFIMAC) and Instituto Universitario de Ciencia de Materiales 'Nicolás Cabrera' (INC), Universidad Autónoma de Madrid, 28049 Madrid, Spain*

Table of Contents

I. Experimental

S1. Synthesis…………………………………………………………...…3

S2. MCBJ conductance measurements…………………………….…….30

S3. STM conductance measurements……………………...………….31

S4. Apparent stretching length…………………………...……………… .32

S5. Clustering analysis……………………………...……………… .33

S6. High-conductance plateau…………………….…………….……….34

S7. Low-conductance plateau…………………………………………35

S8. STM Seebeck coefficient measurements…………………………37

II. Theoretical

S9. Four-site model………………………………………..…………40

S10. Single-level model…………………………………………….....45

References…………………………………...……………...….46

I. Experimental

S1. Synthesis

General Procedures: All commercially available chemicals were used without further purification. Dry solvents were used as crown cap and purchased from Acros Organics and Sigma-Aldrich. NMR solvents were obtained from Cambridge Isotope Laboratories, Inc. (Andover, MA, USA) or Sigma-Aldrich. All NMR experiments were performed on Bruker Avance III or III HD, two- or four-channel NMR spectrometers operating at 400.13, 500.13 or 600.27 MHz proton frequency. The instruments were equipped with direct observe BBFO, indirect BBI or cryogenic four-channel QCI (H/C/N/F) 5 mm probes, all with self-shielded z-gradient. The experiments were performed at 298 K or 295 K. All chemical shifts (δ) are reported in parts per million (ppm) relative to the used solvent and coupling constants (J) are given in Hertz (Hz). The multiplicities are written as: s = singlet, d = doublet, t = triplet, dd = doublet of doublet, m = multiplet. Gas chromatography (GC-MS) was performed on a Shimadzu GC-MS-QP2010 SE gas chromatograph system, with a ZB-5HT inferno column (30 m × 0.25 mm × 0.25 mm), at 1 mL/min He-flow rate (split = 20:1) with a Shimadzu mass detector (EI 70 eV). Flash column chromatography (FCC) was performed with SiliaFlash® P60 from SILICYCLE with a particle size of 40-63 µm (230-400 mesh), and for TLC silica gel 60 F254 glass plates with a thickness of 0.25 mm from Merck were used. The detection was carried out with a UV-lamp at 254 or 366 nm. Gel permeation chromatography (GPC) was performed on a Shimadzu Prominence System with PSS SDV preparative columns from PSS (2 columns in series: 600 mm × 20.0 mm, 5 µm particles, linear porosity "S", operating ranges: 100-100.000 g mol^{-1}) using chloroform as solvent. For HPLC a Shimadzu LC-20AD and a LC-20AT HPLC were used, equipped with a diode array UV/Vis detector (SPD-M10A VP from Shimadzu, λ = 200-600 nm) and a column oven Shimadzu CTO-20AC for analytical

measurements. The used column was a Reprosil 100 C18, 5 µm, 250 × 16 mm; Dr. Maisch GmbH. For preparative HPLC a Shimadzu LC-20Ap was used, equipped with a diode array UV/Vis detector (SPD-20A from Shimadzu, λ = 200-600 nm). The used column was a Reprosil 100 C18, 10 µm, 250 × 30 mm; Dr. Maisch GmbH. High-resolution mass spectra (HRMS) were measured as HR-ESI-ToF-MS with a Maxis 4G instrument from Bruker or as HR-EI-MS spectrometry with a DFS double-focusing (BE geometry) magnetic sector mass spectrometer (ThermoFisher Scientific, Bremen, Germany). Mass spectra were measured with electron ionization (EI) at 70 eV, solid probe inlet, a source temperature of 200 °C, an acceleration voltage of 5 kV, and a resolution of 10'000. The instrument was scanned between e.g. m/z 300 und 350 at scan rate of 100-200 s/decade in the electric scan mode. Perfluorokerosene (PFK, Fluorochem, Derbyshire, UK) served for calibration.

Synthetic steps to the target structures:

a) Overview:

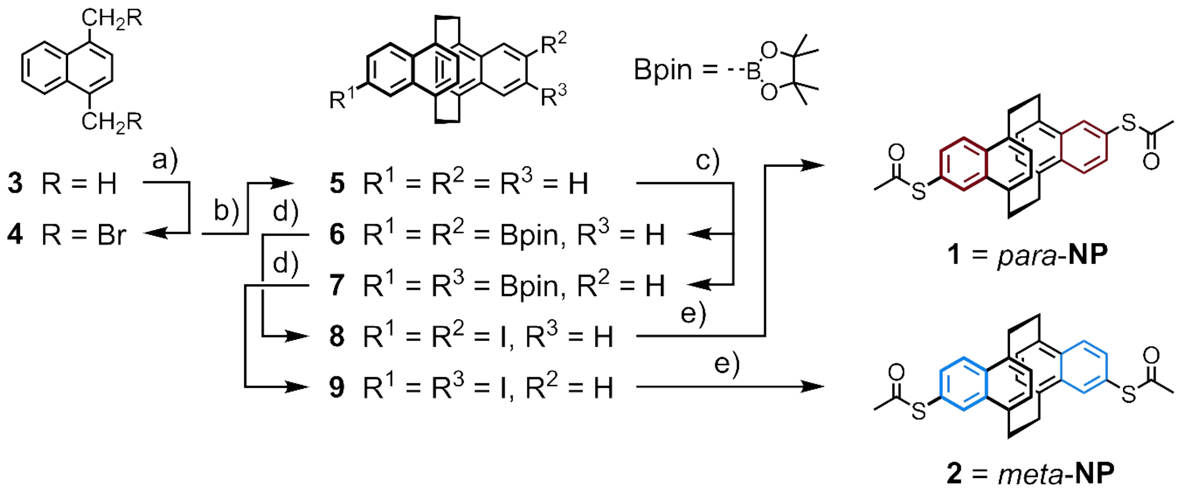

Synthesis of the *anti*-[2.2](1,4)naphthalenophanes, exposing a pair of acetyl protected thiol anchor groups, *para*-**NP** (**1**) and *meta*-**NP** (**2**). Reagents and conditions: a) 3 eq. NBS, 10 mol% $(C_6H_5CO)_2O_2$, CH_2Cl_2, 55 °C, 18 h, 78%; b) 2.5 eq. SmI_2, THF, r.t., 4 h, 89%; c) 1.) 10 mol% $[Ir(COD)(OMe)]_2$, 20 mol% 4,4'-di-*t*Bu-bipy, 2.5 eq. bis(pinacolato)diboron, THF, reflux, 18 h, 77%, as mixture of regioisomers **6** and **7**; 2.) separation of regioisomers by HPLC, isolated yields: 29% of **6** and 31% of **7**; d) 1.) 10 eq. $CH_3B(OH)_2$, CH_2Cl_2, CF_3COOH, r.t., 2 d, evaporation to dryness; 2.) 1 eq. I_2, K_2CO_3, CH_3CN, reflux, 4 h; isolated yields: 65% of **8** and 77% of **9**; e) 4 eq. CH_3COSK, 2 mol% Pd_2dba_3, 4 mol% xantphos, $CH_3C_6H_5/CH_3COCH_3$: 2/1, seal tube, 70° C, 2 h; isolated yields: 69% of **1** and 84% of **2**.

b) Synthetic protocols and characterization:

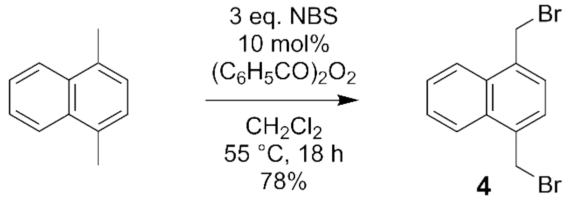

1,4-bis(bromomethyl)naphthalene (4):

Compound **4** was synthesized following a literature known procedure[1]: 1,4-dimethyl-naphthalene (2.00 g, 12.8 mmol, 1.0 eq) was dissolved in dry dichloromethane (80 mL) and degassed with argon. Under an active argon stream, *N*-bromosuccinimide (6.904 g, 38.4 mmol, 3.0 eq) and benzoyl peroxide (413 mg, 1.28 mmol, 10 mol%) were added and the suspension was degassed to give a yellow suspension. The reaction mixture was heated under nitrogen at 55 °C for 18 hours. The reaction mixture was cooled to room temperature and then washed with 2 M HCl (2 x 15 mL), 2 M NaOH (2 x 20 mL), brine, and dried with $MgSO_4$. The solvent was evaporated, and the crude was purified by flash column chromatography (SiO_2, cyclohexane:ethyl acetate 6:1) to yield the desired product in 78% yield (3.124 g, 9.948 mmol).

Analytic data for 4: ^{1}H NMR (500 MHz, $CDCl_3$) δ 8.26 – 8.17 (m, 2H), 7.70 – 7.63 (m, 2H), 7.26 (d, J = 0.8 Hz, 2H), 4.93 (s, 4H). ^{13}C NMR (126 MHz, $CDCl_3$) δ 135.08, 131.75, 127.40, 127.03, 124.75, 31.35. HRMS (ESI-ToF): calcd. for $C_{12}H_{10}Br$ 232.9960 $[M^+]^+$; found 232.9959.

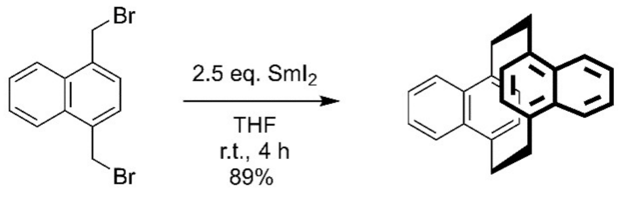

4 5

***anti*-[2.2](1,4)naphthalenophane (5):**

Compound **5** was synthesized following a modification of a literature known procedure[2]: The dibromide compound **3** (1520 mg, 4.84 mmol, 1.0 eq) in THF (40 mL) was added dropwise slowly over 30 minutes to a 0.1 M solution of SmI_2 (5 g in 120 mL, 12.4 mmol, 2.5 eq.) stirred at room temperature under argon. The mixture was then stirred at that temperature for 16 hours, during which time the initial blue color became tinged slightly with green. Subsequently the reaction was slowly quenched with ice, extracted with DCM and the solvent was removed under vacuum. After flash column chromatography (SiO_2, cyclohexane:ethylacetate 3:1) compound **5** (664 mg, 2.153 mmol, 89%) was yielded as a white solid. Note: Small amounts of impurities with similar polarity as the target structure were removed by washing with few mL of chloroform.

Analytic data for 5: ^{1}H NMR (500 MHz, $CDCl_3$) δ 7.84 – 7.61 (m, 4H), 7.51 – 7.35 (m, 4H), 5.76 (s, 4H), 3.95 – 3.55 (m, 4H), 3.26 – 2.82 (m, 4H). ^{13}C NMR (126 MHz, $CDCl_3$) δ 135.03, 134.43, 127.50, 125.24, 124.74, 32.14. HRMS (ESI-ToF): calc. for $C_{24}H_{20}Ag$ 415.0610 [M+Ag]+; found 415.0611.

5,15-bis(4,4,5,5-tetramethyl-1,3,2-dioxaborolan-2-yl)-*anti*-[2.2](1,4)naphthalenophane (6) and 5,16-bis(4,4,5,5-tetramethyl-1,3,2-dioxaborolan-2-yl)-*anti*-[2.2](1,4)-naphthalenophane (7):

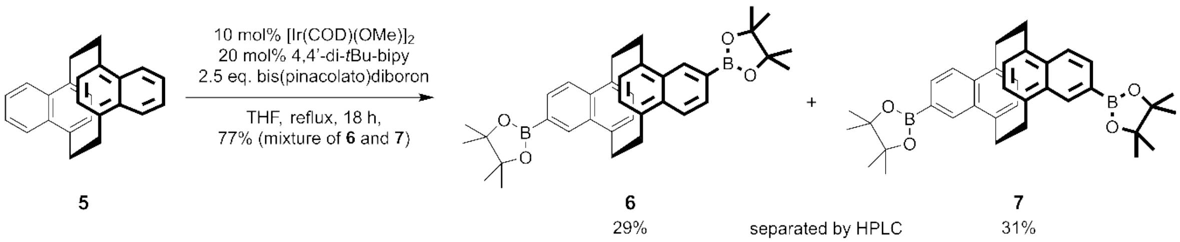

Compounds **6** and **7** were synthesized following a literature known procedure to borylate naphthalenes[3]. A mixture of $[Ir(COD)(OMe)]_2$ (107 mg, 162 μmol, 10 mol%), 4,4'-di-tert-butyl-2,2'-bipyridine (87 mg, 324 μmol, 20 mol%), bis(pinacolato)diboron (987 mg, 3.89 mmol, 2.4 eq) and compound **5** (500 mg, 1.62 mmol, 1.0 eq) in dry and degassed THF (160 mL) was refluxed for 18 hours. Then the reaction was cooled down, the solvent was removed under vacuum, and after flash column chromatography (SiO_2, cyclohexane:ethylacetate 1:3) the mixture of pseudo-*para* and pseudo-*meta* regioisomers **6** and **7** (350 mg, 625 μmol, 77%) was yielded as a white solid. The regioisomers were isolated by preparative HPLC using a normal phase column and *n*-heptane:etylacetate (90:10) as eluent. The ratio of the pseudo-*para* and pseudo-*meta* isomers based on the HPLC chromatogram is 1:1 (see Fig. S1). Due to the low solubility of the sample, several rounds of HPLC were necessary. Starting with half of the crude, after 20 HPLC rounds, 130 mg (0.23 mmol, 29%) of **6** and 142 mg (0.25 mmol, 31%) of **7** were isolated.

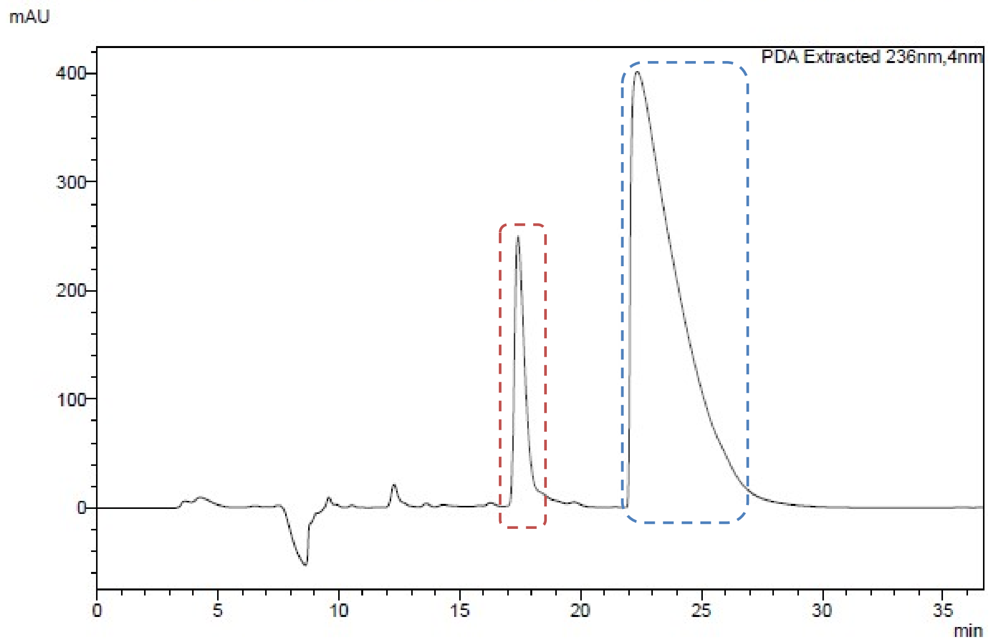

Figure S1: HPLC chromatogram of reversed stationary phase (C18, 1 mL min^{-1}, 90:10 *n*-heptane:ethylacetate, 25 °C) showing the isomeric separation of the regioisomers **6** (orange) and **7** (blue).

Analytic data for 6: ^{1}H NMR (400 MHz, CD_2Cl_2, 22 ºC) δ 8.18 (s, 2H), 7.73 (s, 4H), 5.84 – 5.64 (m, 4H), 3.93 – 3.63 (m, 4H), 3.11 – 2.91 (m, 4H), 1.41 (s, 12H), 1.42 (s, 12H). ^{13}C NMR (126 MHz, CD_2Cl_2, 25 ºC) δ 136.99, 135.81, 134.80, 133.07, 129.86, 128.89, 127.86, 124.61, 84.23, 32.31, 32.25, 25.21, 25.18. HRMS (ESI-ToF): calc. for $C_{36}H_{42}B_2O_4Ag$ 667.2329 [M+Ag]$^+$; found 667.2316.

Analytic data for 7: ^{1}H NMR (500 MHz, CD_2Cl_2, 25 ºC) δ 8.20 (s, 2H), 7.73 (s, 4H), 5.89 – 5.58 (m, 4H), 3.90 – 3.80 (m, 2H), 3.79 – 3.68 (m, 2H), 3.11 – 2.91 (m, 4H), 1.42 (s, 12H), 1.42 (s, 12H). ^{13}C NMR (126 MHz, CD_2Cl_2, 25 ºC) δ 136.99, 135.82, 134.78, 134.68, 133.12, 129.86, 128.76, 127.96, 124.61, 84.23, 32.42, 32.15, 25.22, 25.18. HRMS (ESI-ToF): calc. for $C_{36}H_{42}B_2O_4Na$ 583.3167 [M+Na]$^+$; found 583.3161.

5,15-bis(iodo)-*anti*-[2.2](1,4)naphthalenophane (8) and 5,16-bis(iodo)-*anti*-[2.2](1,4)-naphthalenophane (9):

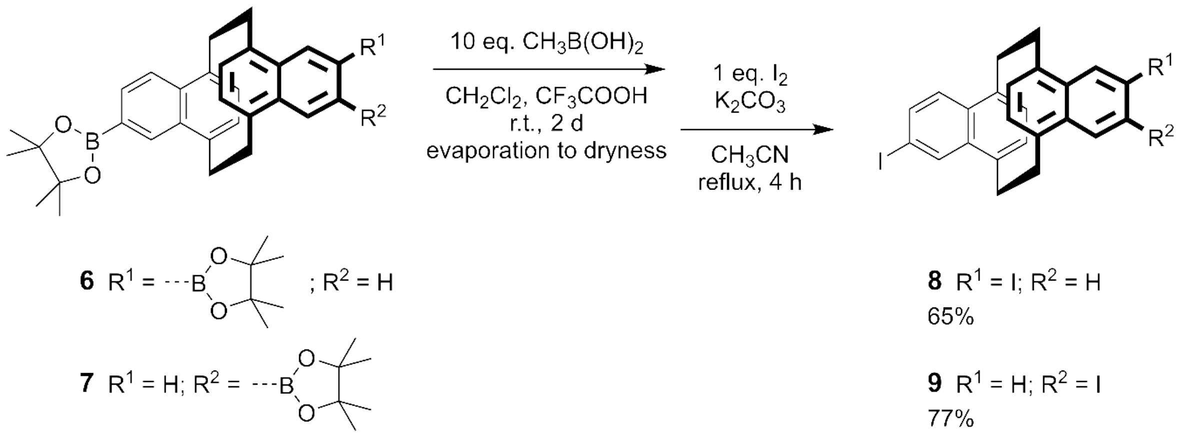

Compounds **8** and **9** were synthesized by the initial hydrolysis of the boronic esters followed by the substitution with iodine. The bis(pinacolato)diboron compounds (**6** and **7** (100 mg, 171 mmol, 1 eq)) were placed, separately, in a 100 mL round-bottom flask together with 10 eq of methylboronic acid (103 mg, 1.71 mol) and dissolved in 10 mL of methylene chloride. Then trifluoroacetic acid (100 μL) was added, and the mixture was stirred at room temperature for 2 days. Afterwards all volatiles were evaporated, and the crude residue was used for the next step of the synthesis without further purification.

The crude sample of substituted arylboronic acids and K_2CO_3 was added to a 20 mL round-bottomed flask equipped with a magnetic stirring bar. The tube was evacuated twice and back-filled with nitrogen. Acetonitrile (2 mL) and iodine (43 mg, 171 mmol) were added to the tube at room temperature under a stream of nitrogen, and the tube was sealed and put into a pre-heated oil bath at 80 °C for 4 h under nitrogen atmosphere. After the resulting solution was cooled to room temperature, $Na_2S_2O_3$ aq. (10 mL) was added to the resulting mixture, and then the aqueous layer was extracted with EtOAc (3 × 5 mL). The combined organic phase was dried over anhydrous Na_2SO_4, filtered and concentrated by rotary evaporation. After flash column chromatography

(SiO_2, cyclohexane) the diiodinated compounds (**8**: 74 mg, 0.111 mmol, 65%; **9**: 88 mg, 0.131 mmol, 77%) were isolated as a white solid.

Analytic data for 8: ^{1}H NMR (500 MHz, $CDCl_3$) δ 8.08 (d, J = 1.8 Hz, 2H), 7.67 (dd, J = 8.7, 1.8 Hz, 2H), 7.45 (d, J = 8.7 Hz, 2H), 5.94 – 5.76 (m, 4H), 3.77 – 3.59 (m, 4H), 3.06 – 2.86 (m, 4H). ^{13}C NMR (126 MHz, $CDCl_3$) δ 136.83, 134.80, 134.18, 133.58, 133.46, 128.49, 128.46, 126.94, 90.88, 32.02. HRMS (ESI-ToF): calc. for $C_{24}H_{18}I_2Ag$ 666.8543 $[M+Ag]^+$; found 666.8534.

Analytic data for 9: ^{1}H NMR (500 MHz, $CDCl_3$) δ 8.09 (d, J = 1.8 Hz, 2H), 7.67 (dd, J = 8.7, 1.8 Hz, 2H), 7.44 (d, J = 8.7 Hz, 2H), 5.87 – 5.81 (m, 4H), 3.74 – 3.61 (m, 4H), 3.06 – 2.90 (m, 4H). ^{13}C NMR (126 MHz, $CDCl_3$) δ 136.80, 134.69, 134.18, 133.79, 133.69, 133.44, 128.65, 128.30, 126.94, 90.88, 32.05, 31.98. HRMS (ESI-ToF): calc. for $C_{24}H_{18}I_2Ag$ 666.8543 $[M+Ag]^+$; found 666.8537.

5,15-bis(acetylthio)-*anti*-[2.2](1,4)naphthalenophane (1) and 5,16-bis(acetylthio)-*anti*-[2.2](1,4)naphthalenophane (2):

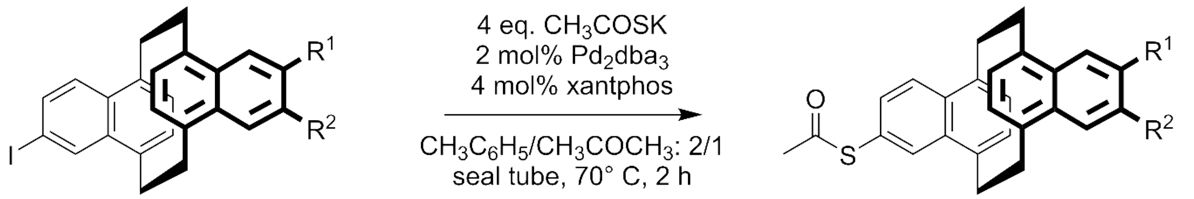

8 R^1 = I; R^2 = H

9 R^1 = H; R^2 = I

1 R^1 = $SCOCH_3$; R^2 = H 69%

2 R^1 = H; R^2 = $SCOCH_3$ 84%

Substituted diiodo compounds (**8** and **9** (70 mg, 105 mmol, 1 eq)) were placed, separately, with 4,5-bis(diphenylphosphino)-9,9-dimethylxanthene (Xantphos, 243 mg, 0.42 mmol) and suspended

in 2:1 toluene/acetone (17 mL). The mixture was sparged with argon, then treated with tris(dibenzylideneacetone)dipalladium(0) (Pd_2dba_3, 192 mg, 0.21 mmol) and potassium ethanethioate (50 mg, 438 mmol, 4 eq). The reaction mixture was sealed in a tube, stirred vigorously, and heated to 70 °C for 2 hours. The solvents were removed under reduced pressure and after flash column chromatography (SiO_2, cyclohexane/ethyl acetate 1:1) compounds **1** (33 mg, 0.072 mmol, 69%) and **2** (40 mg, 0.088 mmol, 84%) were isolated as white solids.

Analytic data for 1: ^{1}H NMR (500 MHz, $CDCl_3$) δ 8.08 (d, J = 1.8 Hz, 2H), 7.67 (dd, J = 8.7, 1.8 Hz, 2H), 7.45 (d, J = 8.7 Hz, 2H), 5.94 – 5.76 (m, 4H), 3.77 – 3.59 (m, 4H), 3.06 – 2.86 (m, 4H). ^{13}C NMR (126 MHz, $CDCl_3$) δ 136.83, 134.80, 134.18, 133.58, 133.46, 128.49, 128.46, 126.94, 90.88, 32.02. HRMS (ESI-ToF): calc. for $C_{28}H_{24}O_2S_2Na$ 479.1110 $[M+Na]^+$; found 479.1102.

Analytic data for 2: ^{1}H NMR (500 MHz, $CDCl_3$) δ 7.82 (d, J = 1.8 Hz, 2H), 7.76 (d, J = 8.5 Hz, 2H), 7.40 (dd, J = 8.5, 1.8 Hz, 2H), 6.16 (d, J = 7.1 Hz, 2H), 5.97 (d, J = 7.1 Hz, 2H), 3.81 – 3.66 (m, 4H), 3.17 – 2.97 (m, 4H), 2.56 (s, 6H). ^{13}C NMR (126 MHz, $CDCl_3$) δ 194.71, 135.31, 135.00, 134.85, 134.39, 132.31, 129.78, 129.13, 128.99, 126.05, 124.07, 32.19, 32.09, 30.52. HRMS (ESI-ToF): calc. for $C_{28}H_{25}O_2S_2$ 457.1290 $[M+H]^+$; found 457.1286.

^{1}H-, ^{13}C-NMR ($CDCl_3$, 400/101 MHz, 25 °C) and HR-ESI-MS spectra of compound 4:

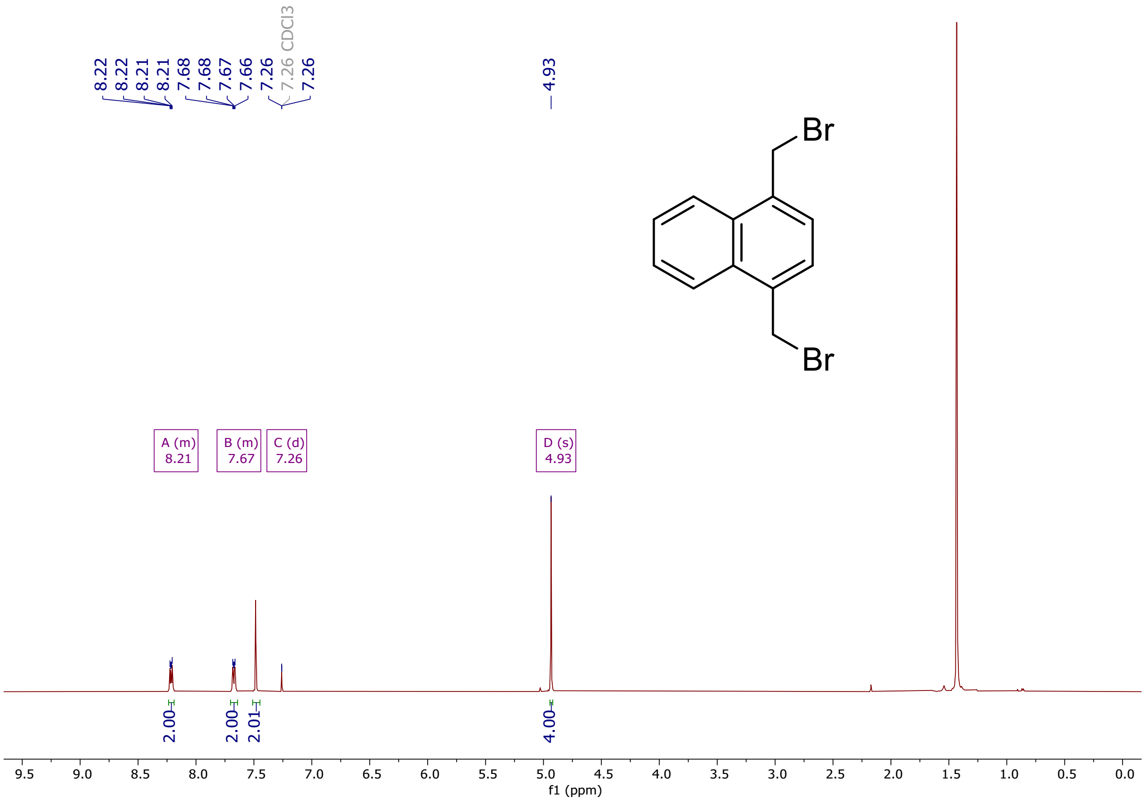

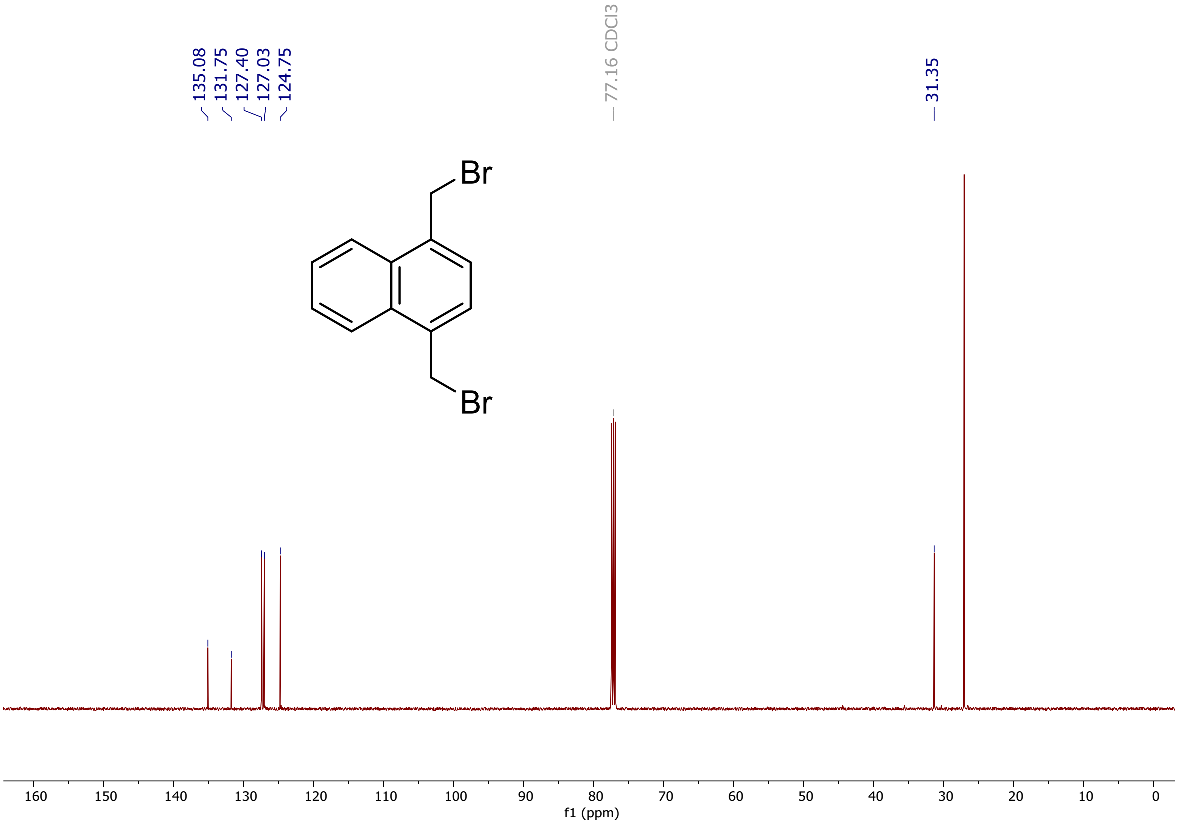

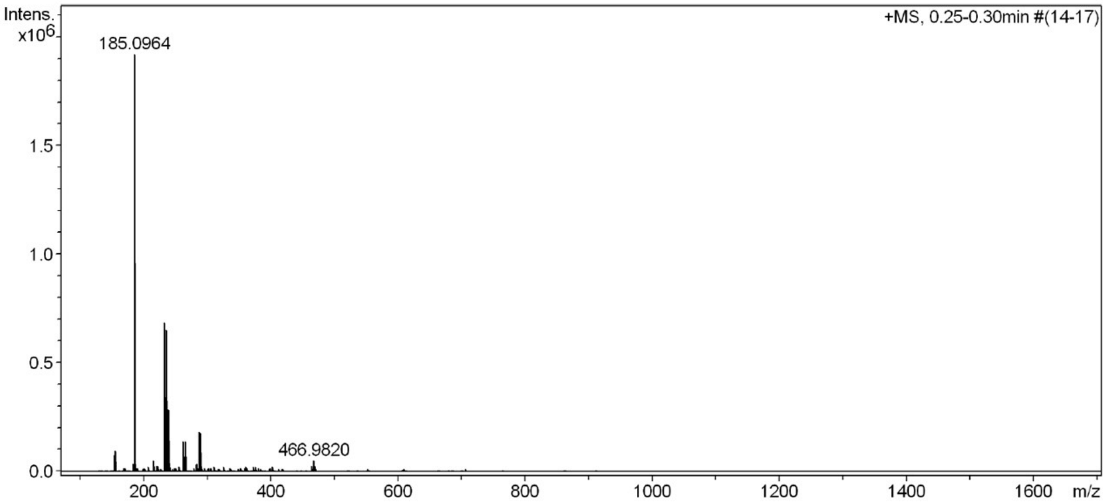

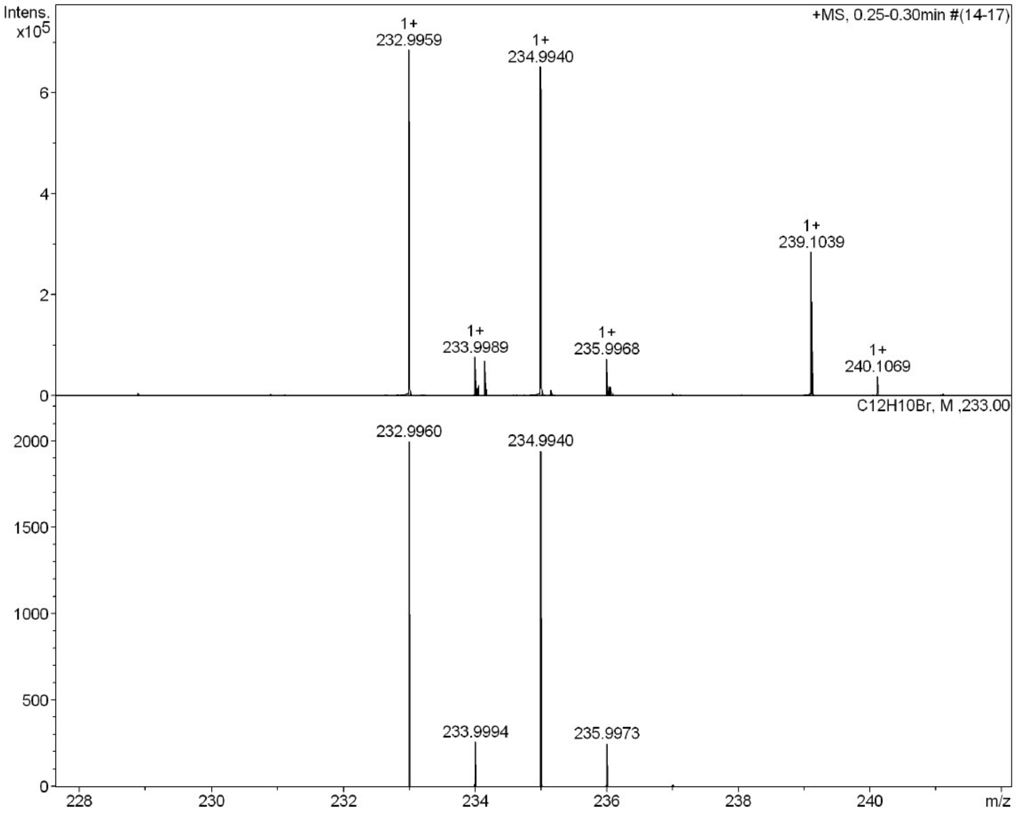

Measured m/z vs. theoretical m/z

Meas. m/z	#	Formula	Score	m/z	err [mDa]	err [ppm]	mSigma	rdb	e⁻ Conf	z
232.9959	1	C 12 H 10 Br	100.00	232.9960	0.1	0.4	16.9	7.5	even	1+

^{1}H-, ^{13}C-NMR ($CDCl_3$, 400/101 MHz, 25 °C) and HR-ESI-MS spectra of compound 5:

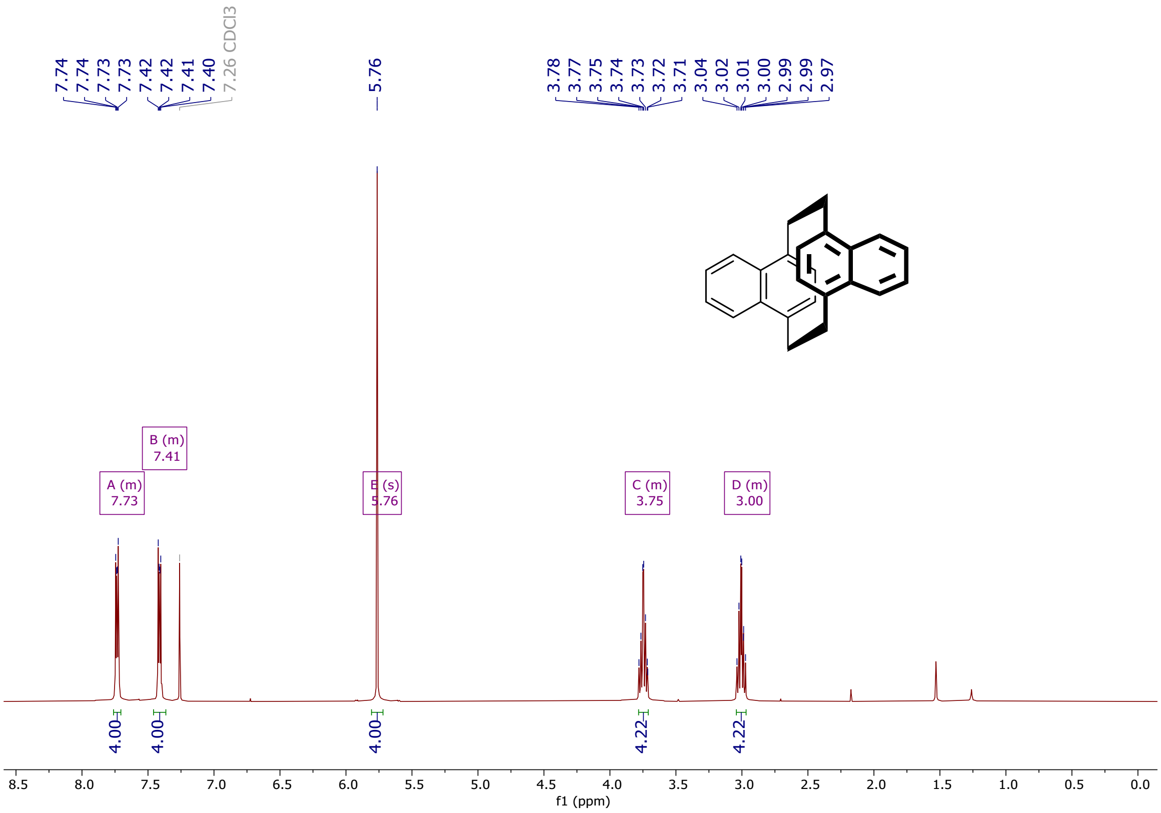

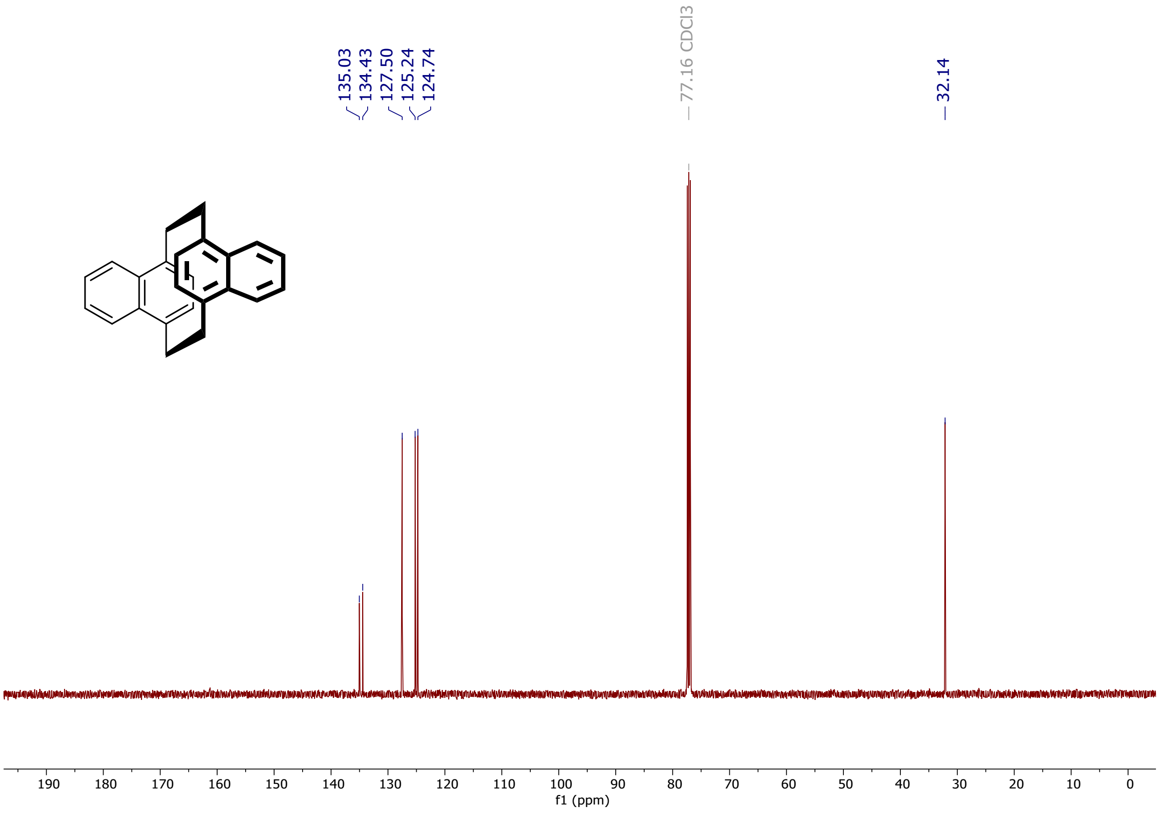

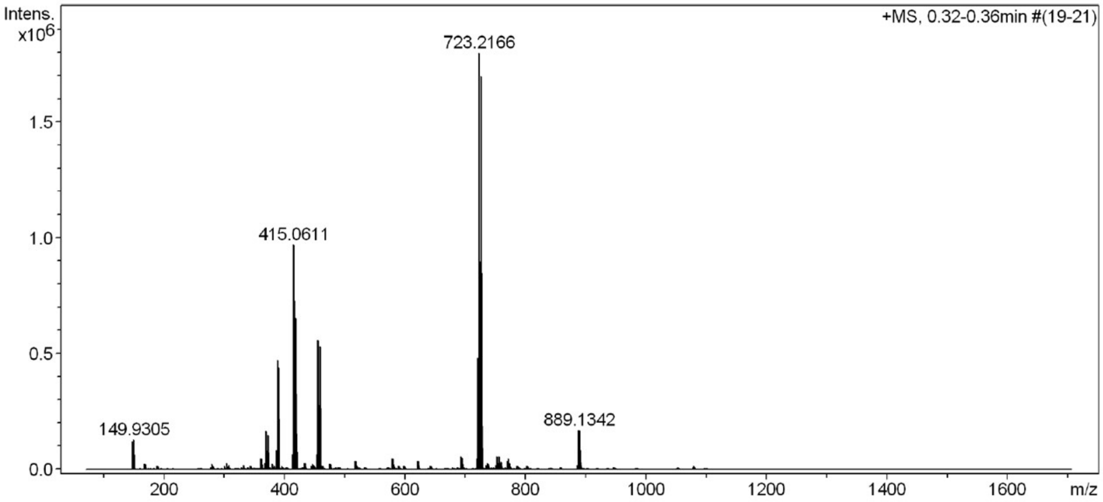

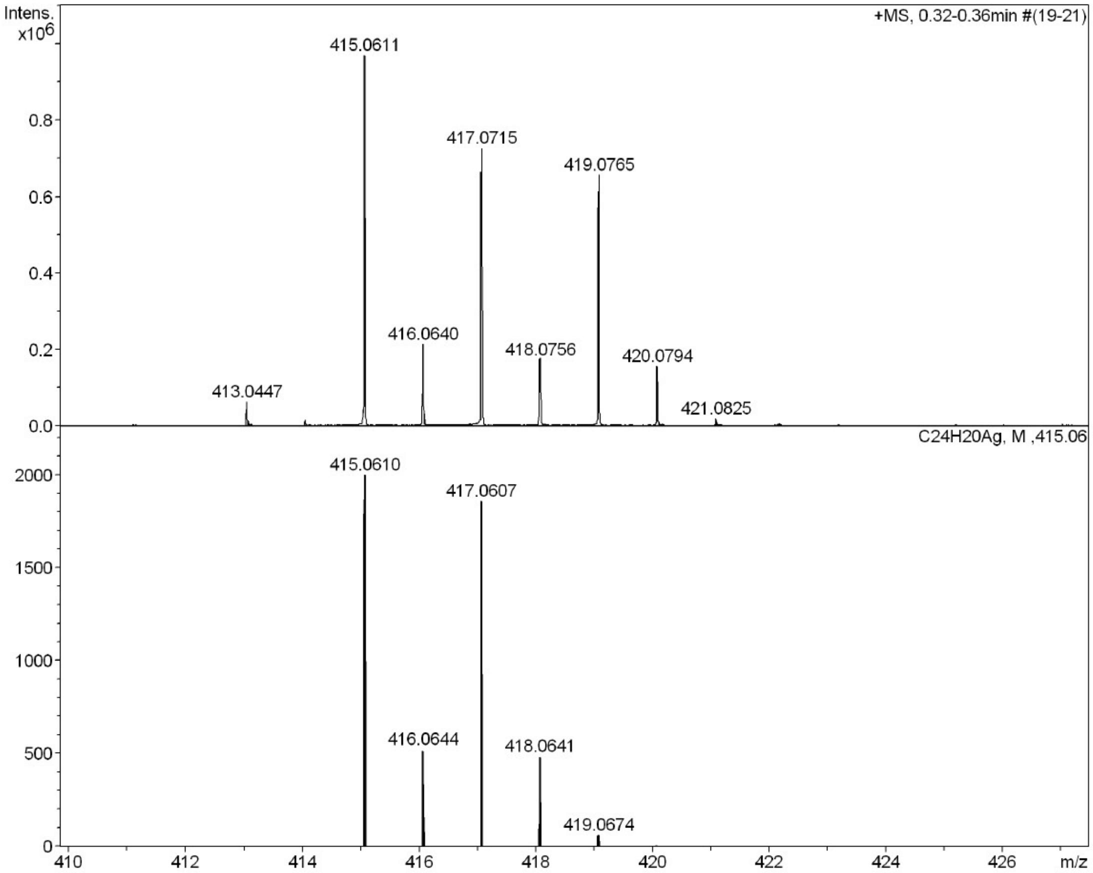

Measured m/z vs. theoretical m/z

Meas. m/z	#	Formula	Score	m/z	err [mDa]	err [ppm]	mSigma	rdb	e¯ Conf	z
415.0611	1	C 24 H 20 Ag	100.00	415.0610	-0.1	-0.2	443.8	14.5	even	1+

^{1}H-, ^{13}C-NMR ($CDCl_3$, 400/101 MHz, 25 °C) and HR-ESI-MS spectra of compound 6:

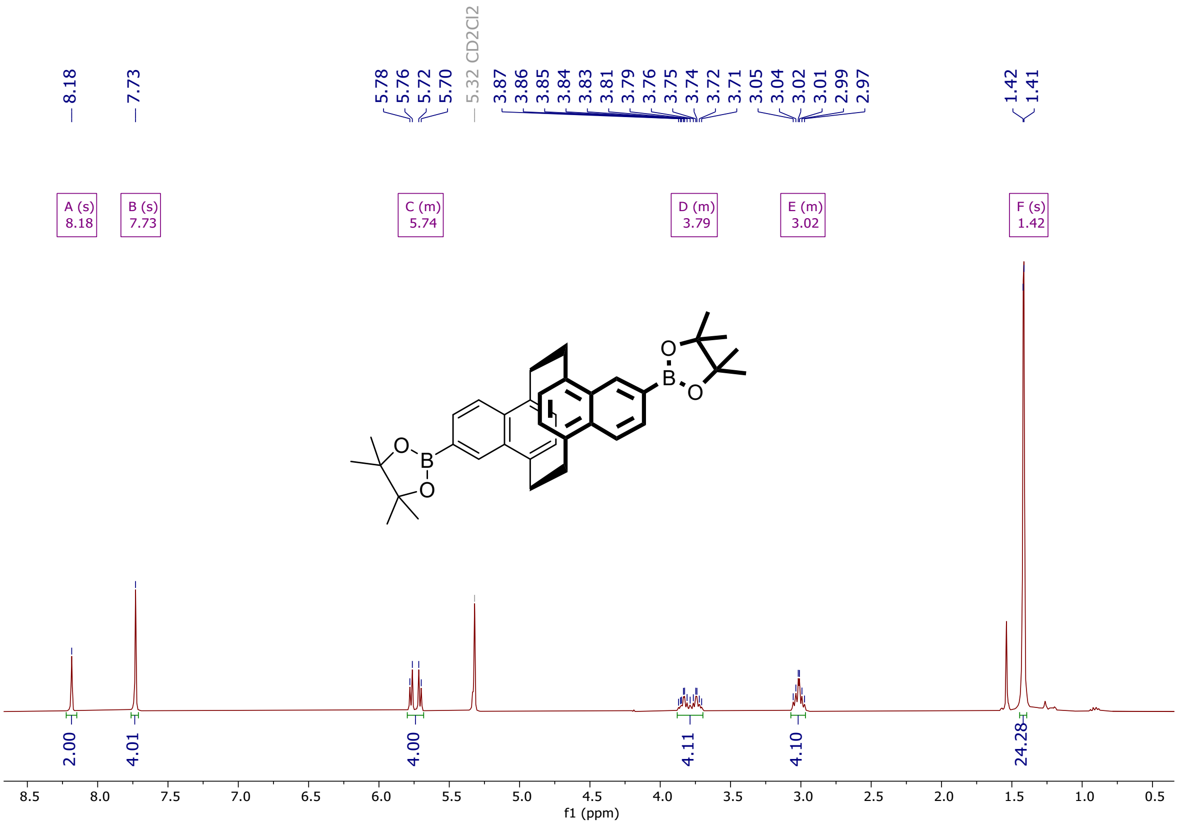

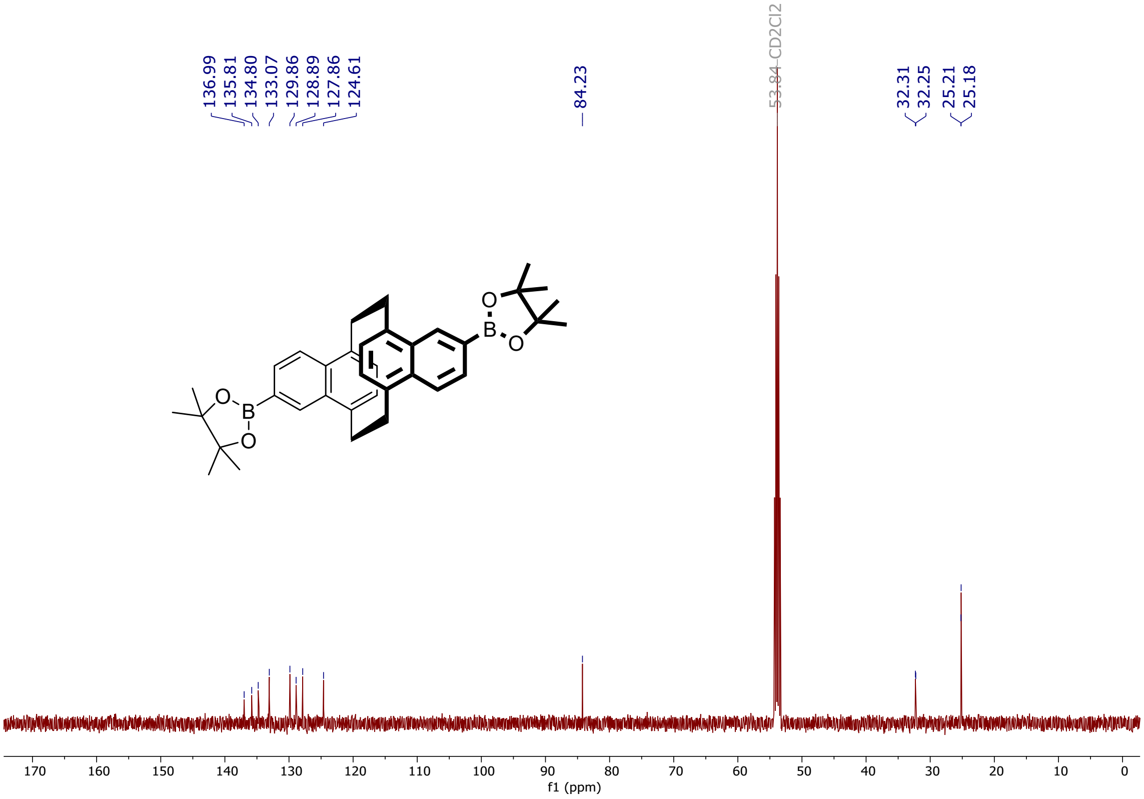

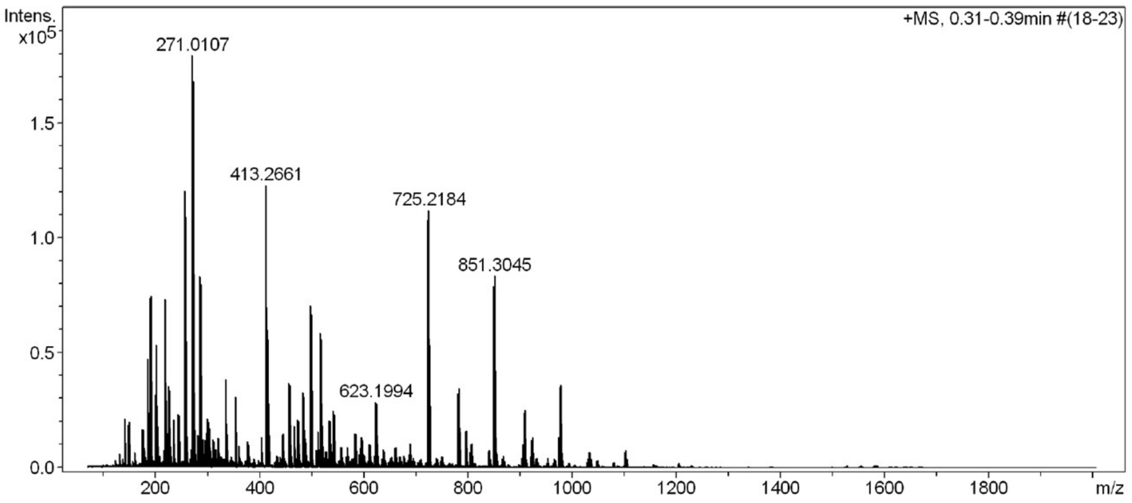

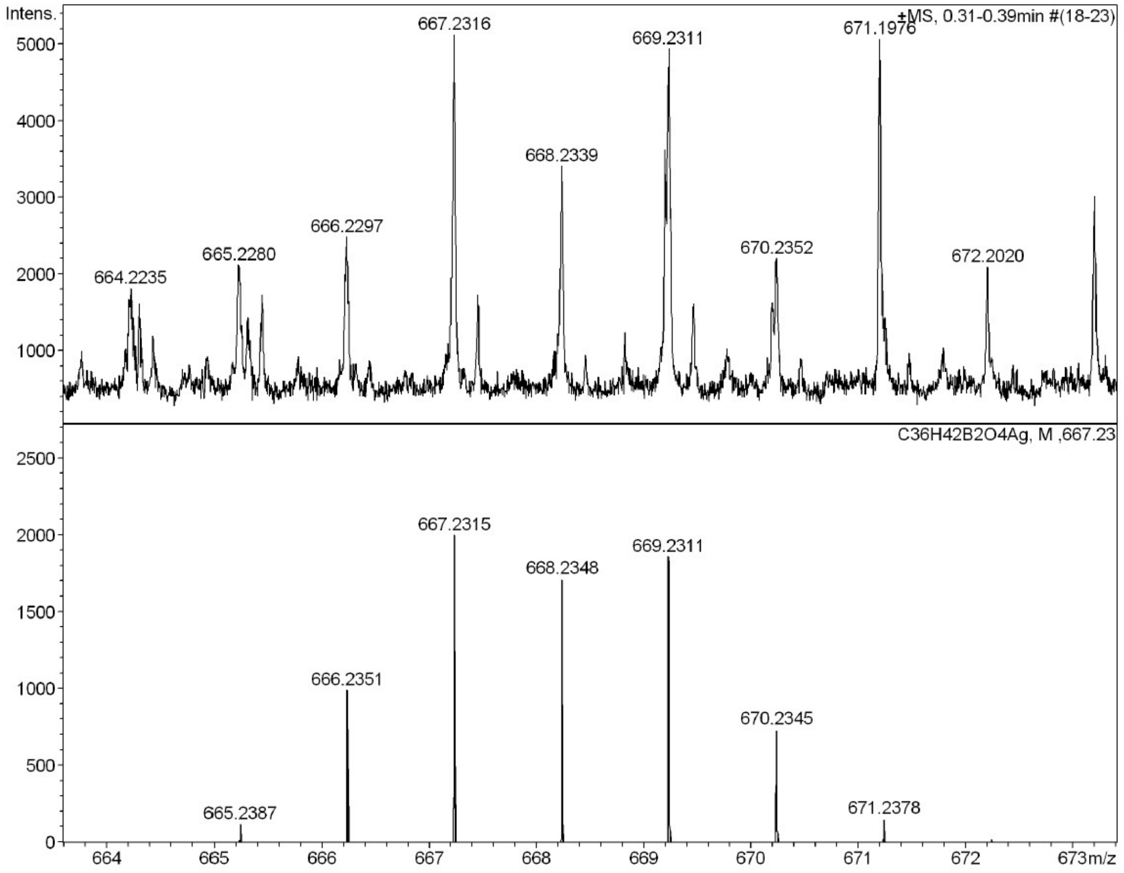

Measured m/z vs. theoretical m/z

Meas. m/z	#	Formula	Score	m/z	err [mDa]	err [ppm]	mSigma	rdb	e⁻ Conf	z
667.2316	1	C 36 H 42 Ag B 2 O 4	100.00	667.2329	-0.2	-0.3	140.0	16.5	even	1+

^{1}H-, ^{13}C-NMR ($CDCl_3$, 400/101 MHz, 25 °C) and HR-ESI-MS spectra of compound 7:

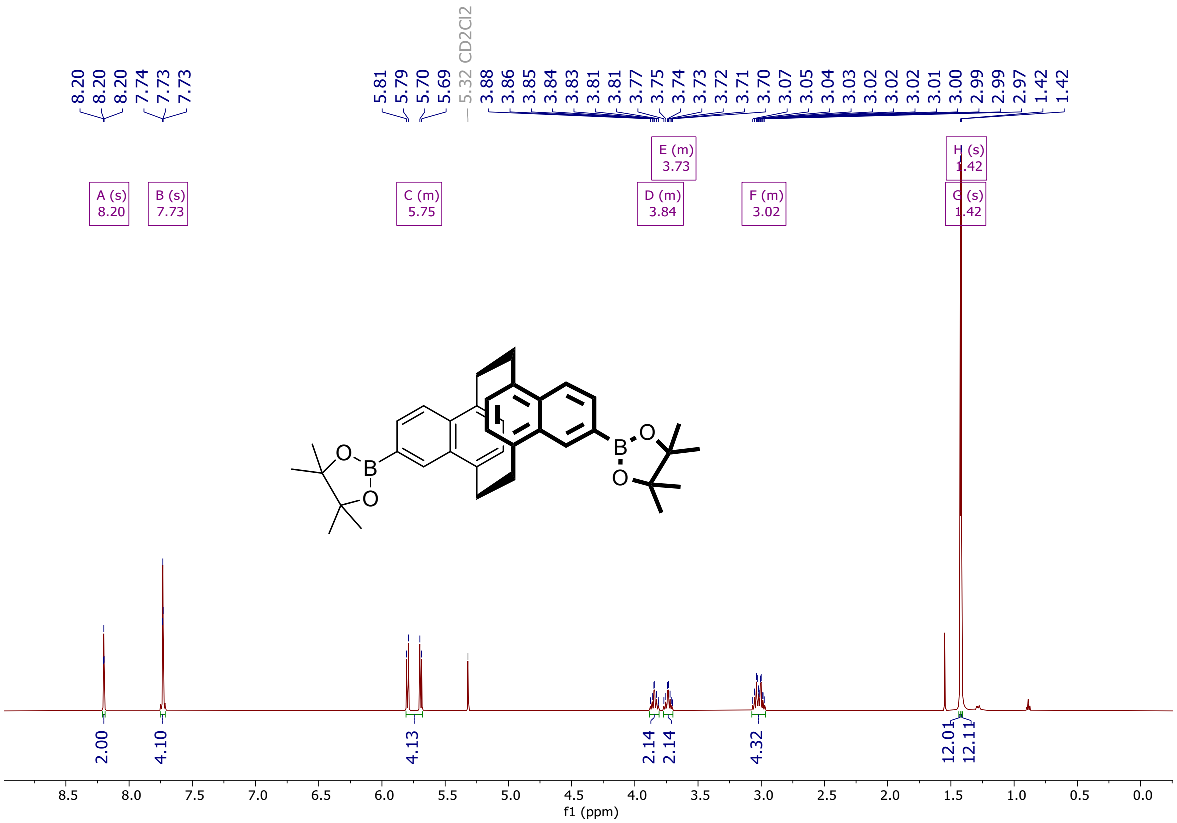

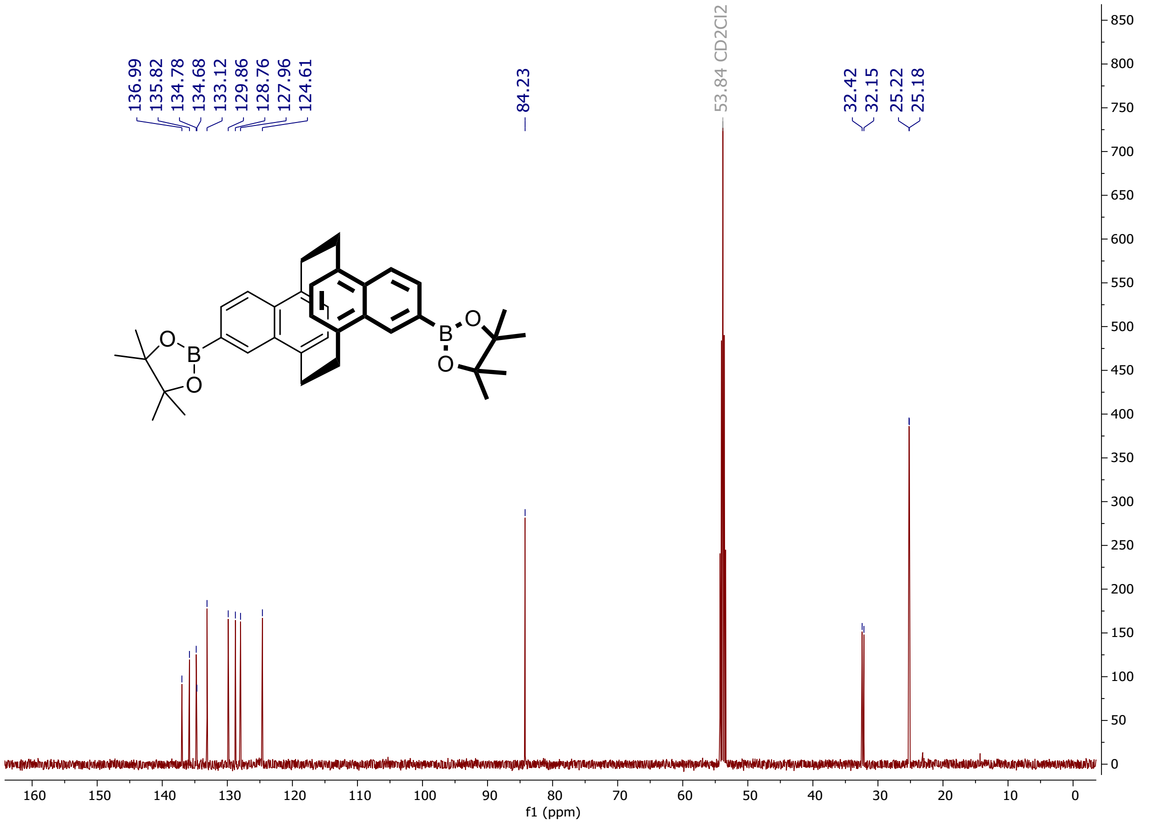
136.99
135.82
134.78
134.68
133.12
129.86
128.76
127.96
124.61
84.23
53.84 CD2Cl2
32.42
32.15
25.22
25.18
f1 (ppm)

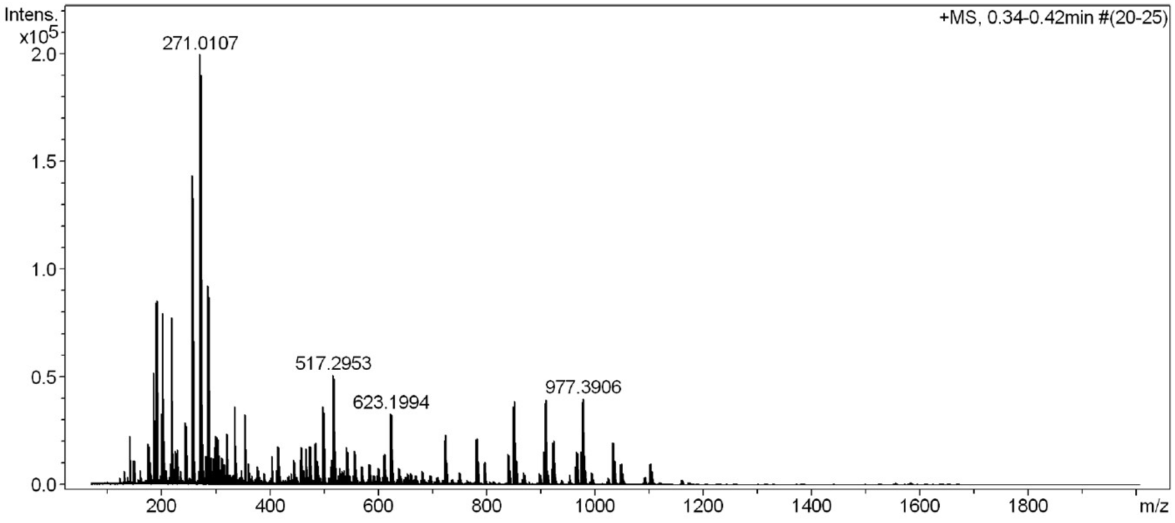
Intens.
x10^5
+MS, 0.34-0.42min #(20-25)
271.0107
517.2953
623.1994
977.3906
m/z

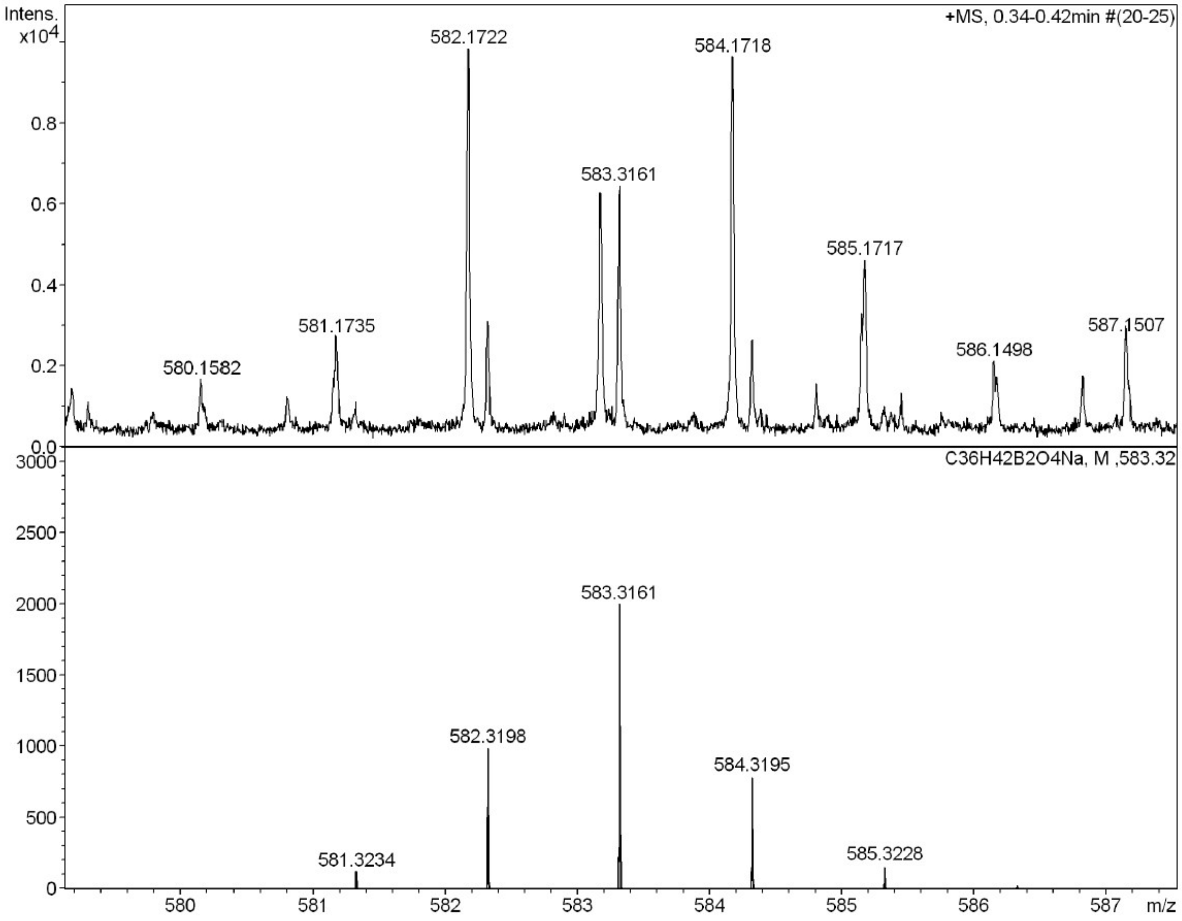

Measured m/z vs. theoretical m/z

Meas. m/z	#	Formula	Score	m/z	err [mDa]	err [ppm]	mSigma	rdb	e⁻ Conf	z

Mass list

#	m/z	I %	I
1	581.3234	6.2	123
2	582.3198	49.7	993
3	582.3268	2.4	48
4	583.3161	100.0	2000
5	583.3231	19.6	392
6	583.3301	0.5	9
7	584.3195	39.7	794
8	584.3265	4.1	82
9	585.3228	8.5	169
10	585.3298	0.6	12
11	586.3262	1.3	26

Acquisition Parameter

**General**	Fore Vacuum	2.39e+000 mBar	High Vacuum	9.68e-008 mBar	Source Type	ESI
	Scan Begin	75 m/z	Scan End	2000 m/z	Ion Polarity	Positive
**Source**	Set Nebulizer	2.0 Bar	Set Capillary	4500 V	Set Dry Gas	8.0 l/min
	Set Dry Heater	200 °C	Set End Plate Offset	-500 V		
**Quadrupole**	Set Ion Energy (MS only)	4.0 eV				
**Coll. Cell**	Collision Energy	8.0 eV	Set Collision Cell RF	600.0 Vpp		

^{1}H-, ^{13}C-NMR ($CDCl_3$, 400/101 MHz, 25 °C) and HR-ESI-MS spectra of compound 8:

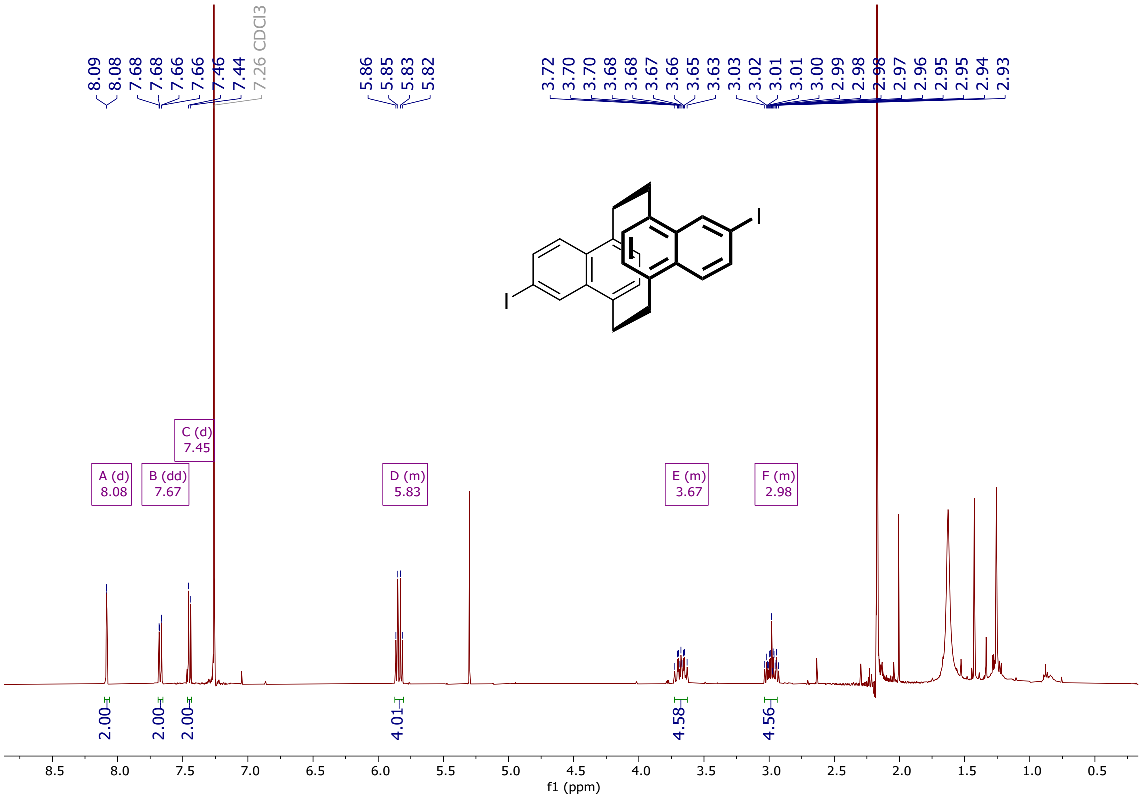

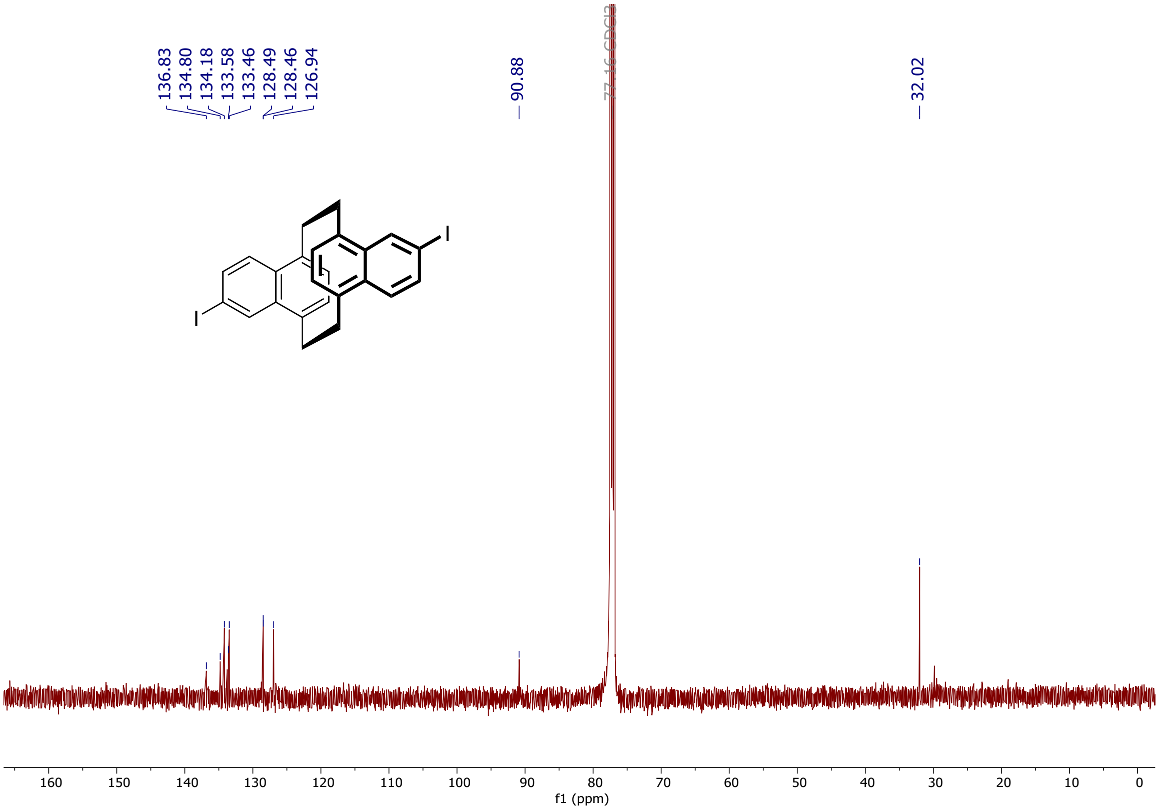

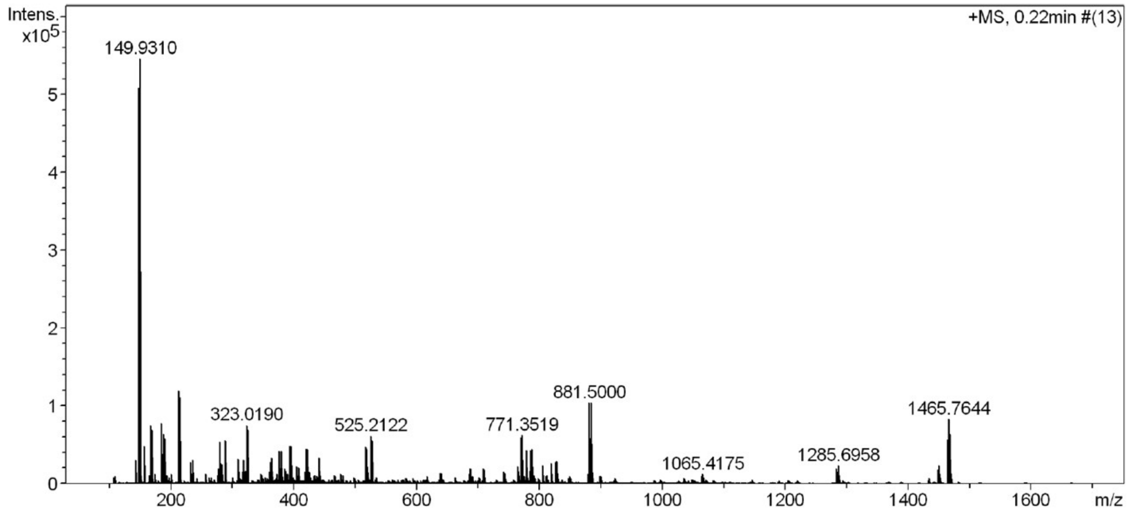

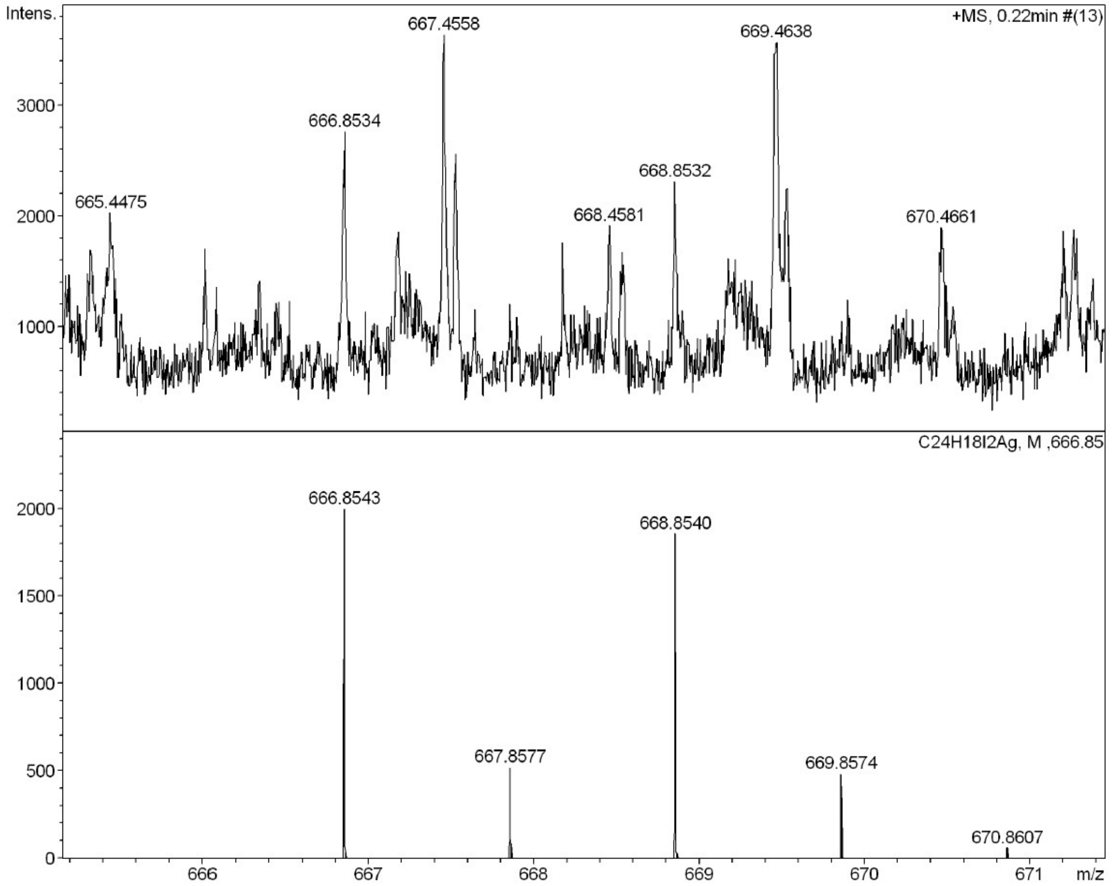

Measured m/z vs. theoretical m/z

Meas. m/z	#	Formula	Score	m/z	err [mDa]	err [ppm]	mSigma	rdb	e⁻ Conf	z
666.8534	1	C 24 H 18 Ag I 2	100.00	666.8543	0.9	1.4	168.6	14.5	even	1+

^{1}H-, ^{13}C-NMR ($CDCl_3$, 400/101 MHz, 25 °C) and HR-ESI-MS spectra of compound 9:

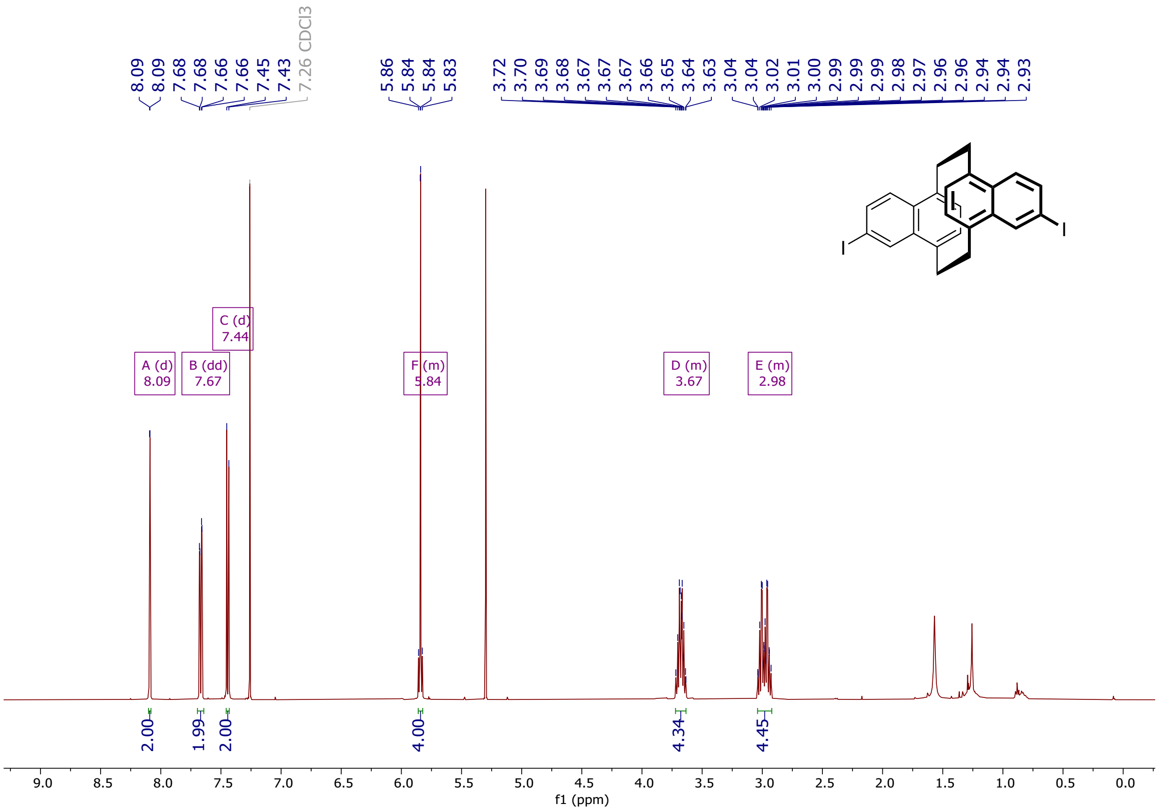

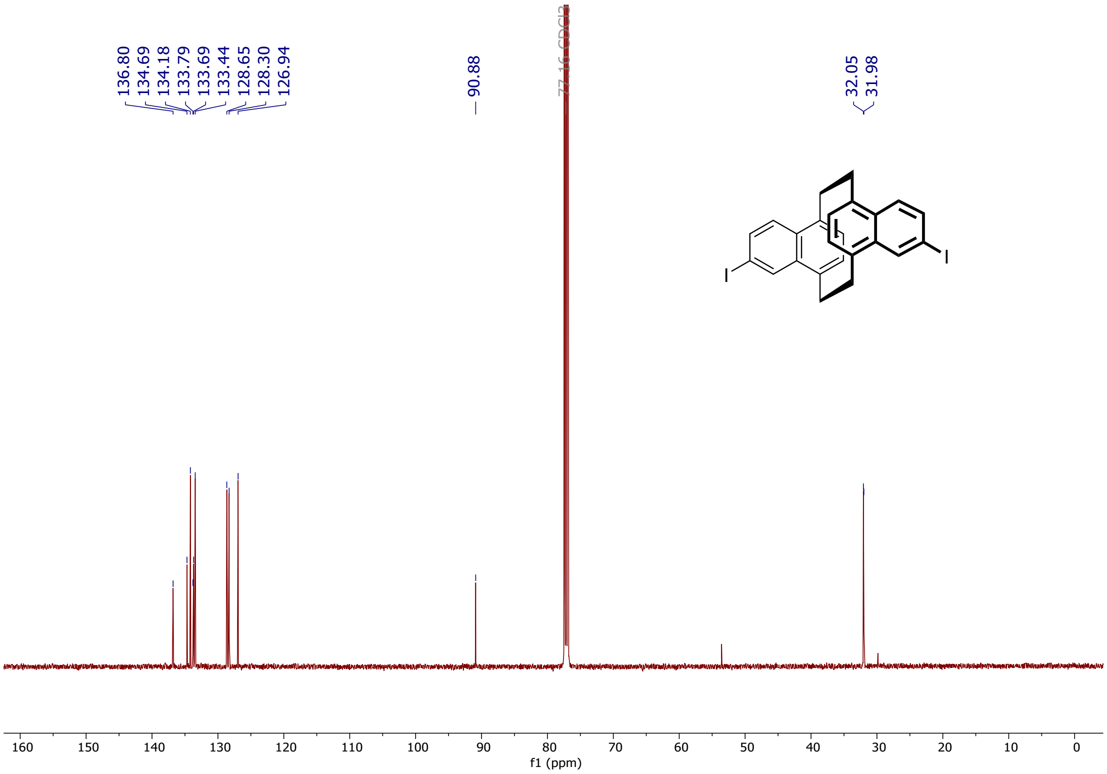

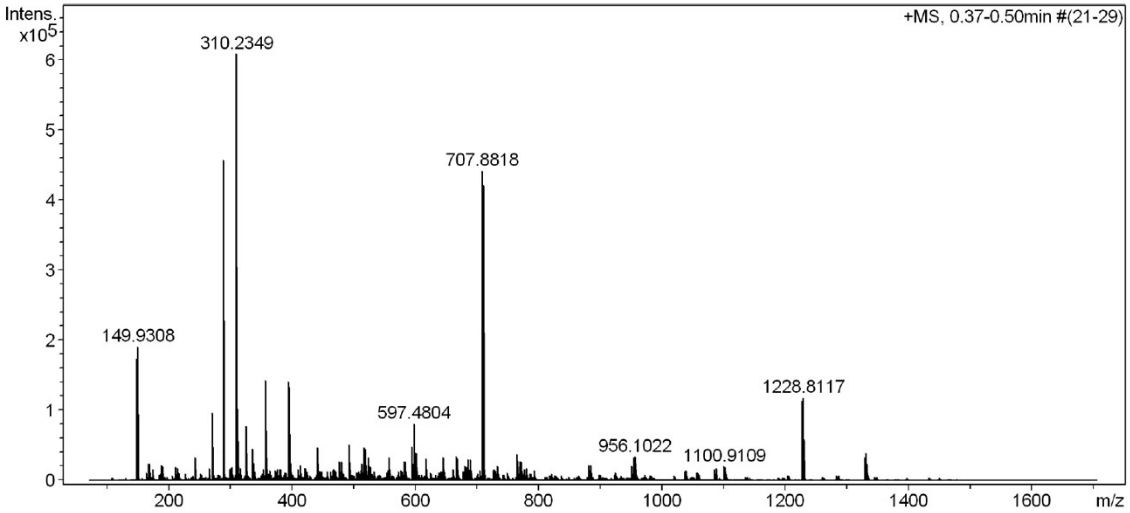

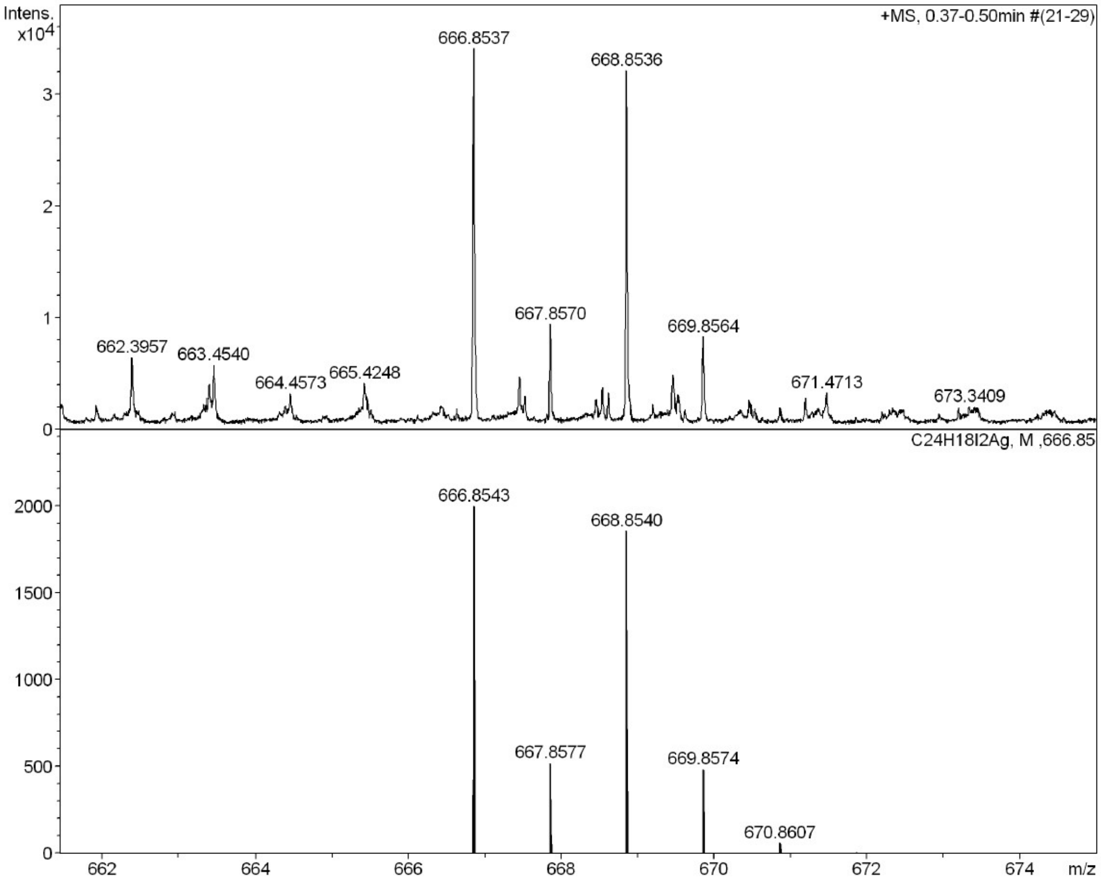

Measured m/z vs. theoretical m/z

Meas. m/z	#	Formula	Score	m/z	err [mDa]	err [ppm]	mSigma	rdb	e⁻ Conf	z
666.8537	1	C 24 H 18 Ag I 2	100.00	666.8543	0.6	0.9	17.4	14.5	even	1+

^{1}H-, ^{13}C-NMR ($CDCl_3$, 400/101 MHz, 25 °C) and HR-ESI-MS spectra of compound 1:

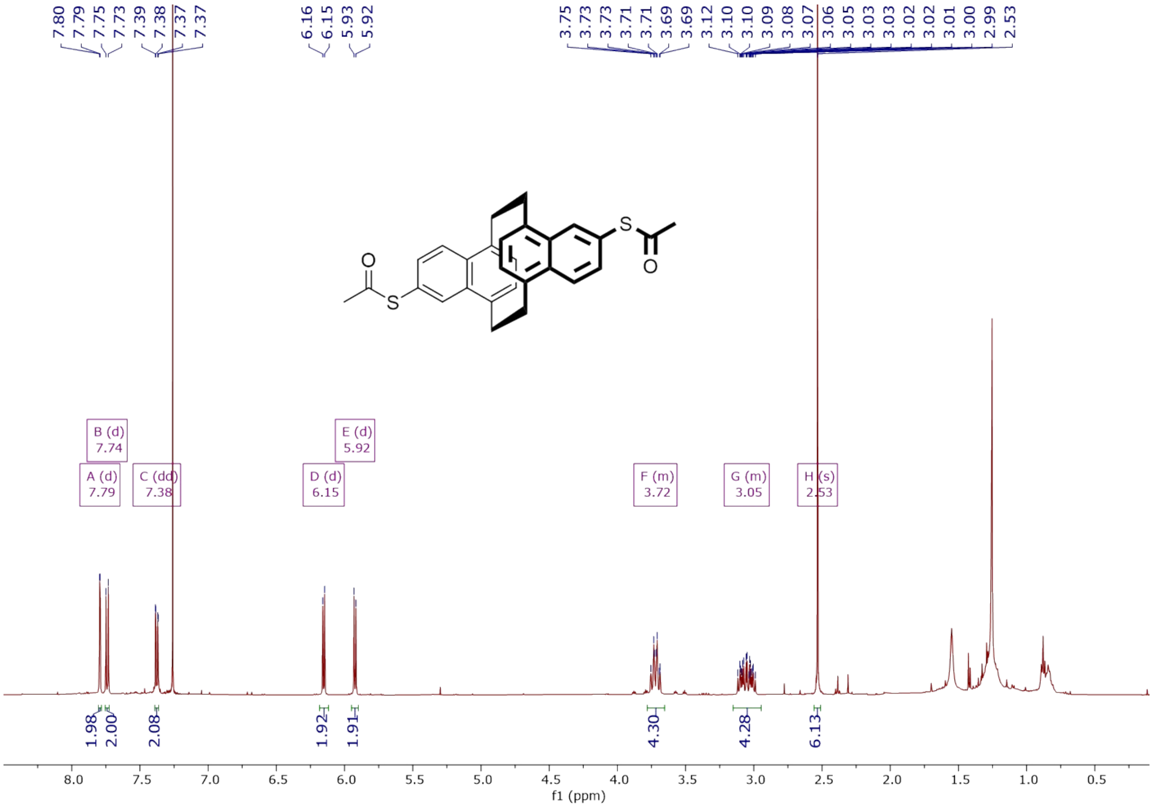

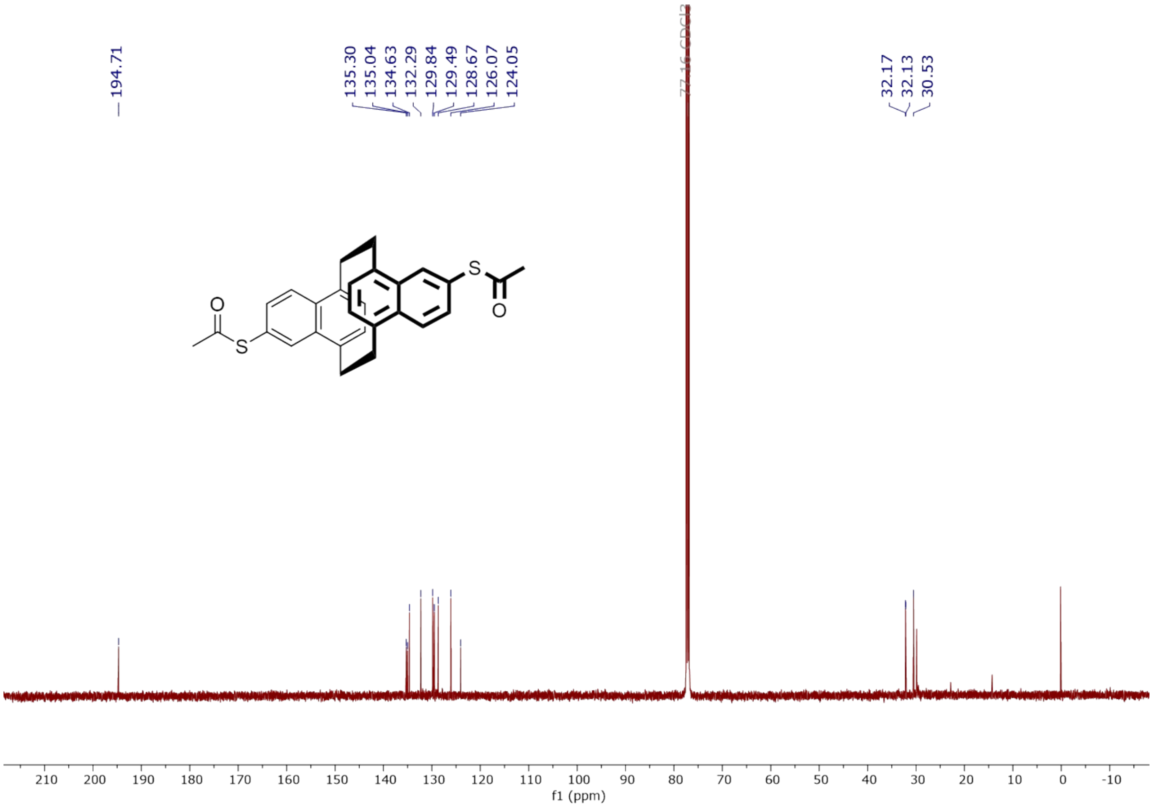

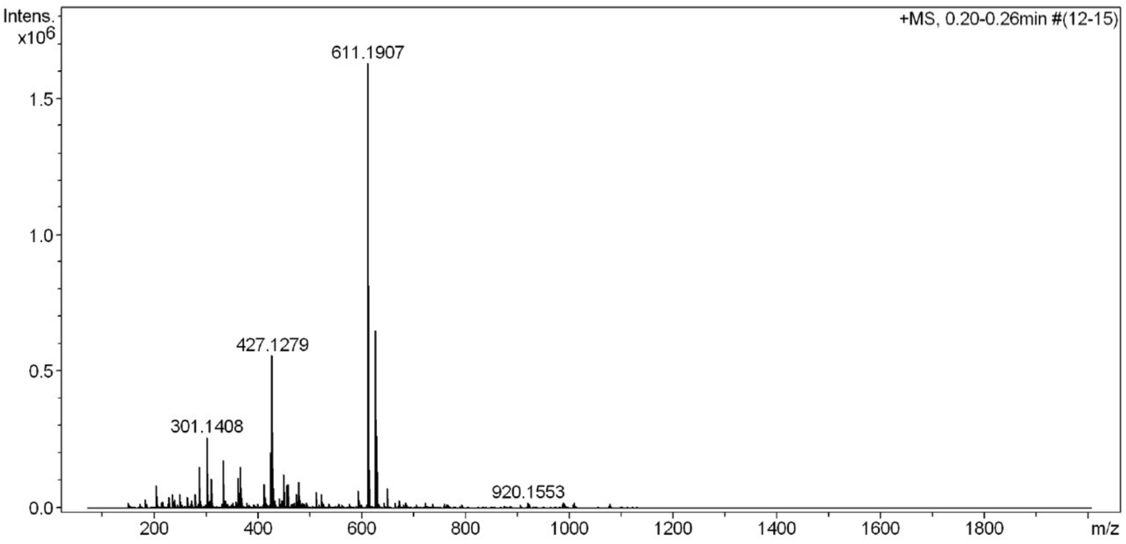

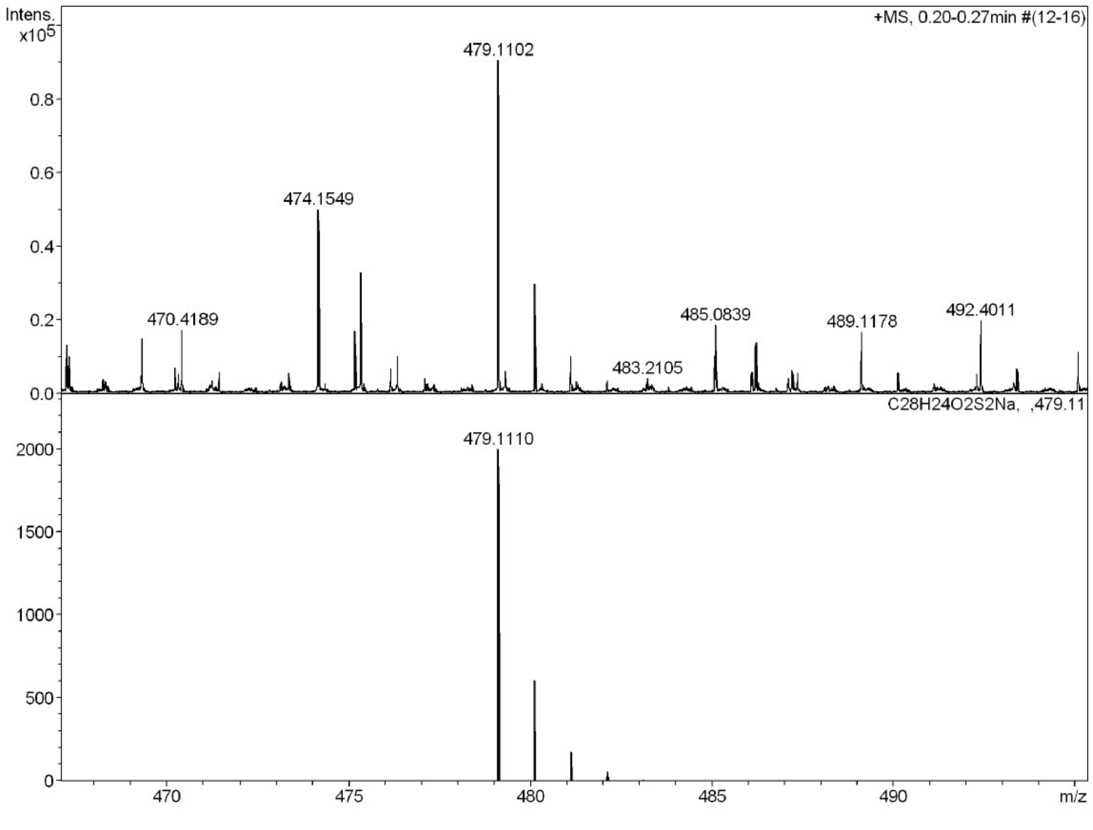

Measured m/z vs. theoretical m/z

Meas. m/z	#	Formula	Score	m/z	err [mDa]	err [ppm]	mSigma	rdb	e⁻ Conf	z
479.1102	1	C 28 H 24 Na O 2 S 2	100.00	479.1110	0.8	1.6	14.8	16.5	even	1+

^{1}H-, ^{13}C-NMR ($CDCl_3$, 400/101 MHz, 25 °C) and HR-ESI-MS spectra of compound 2:

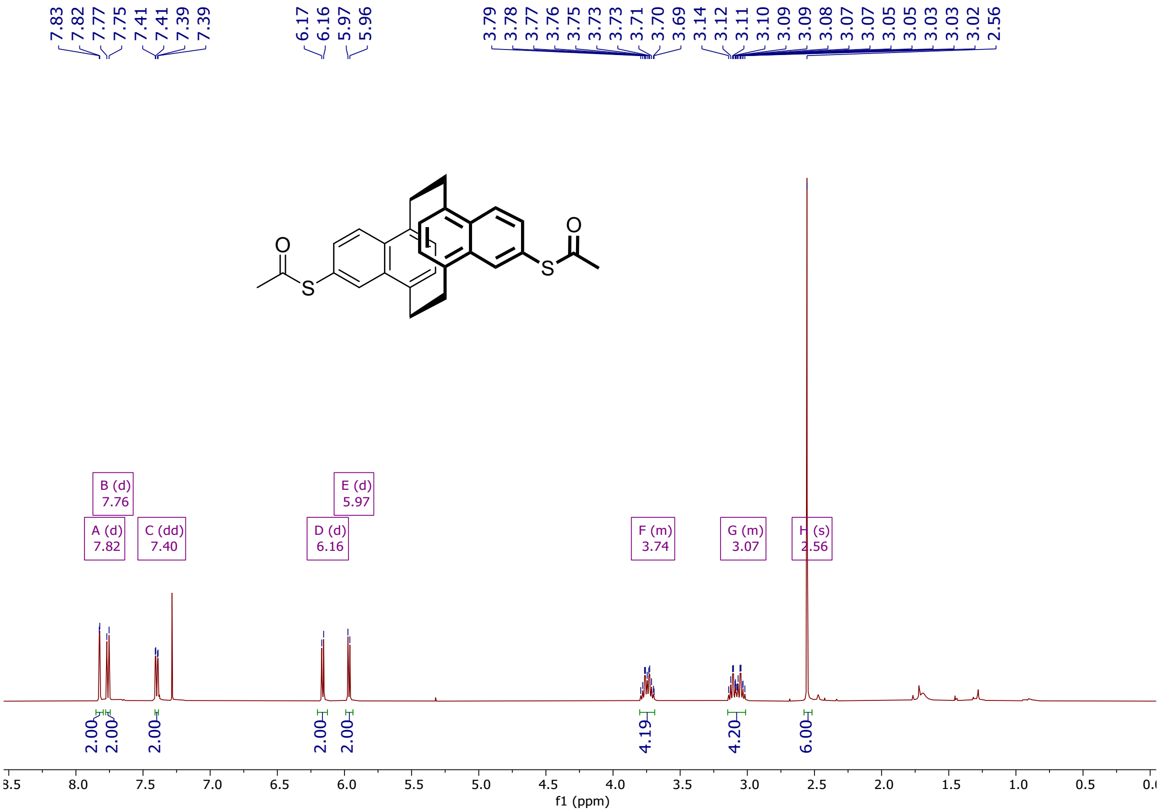

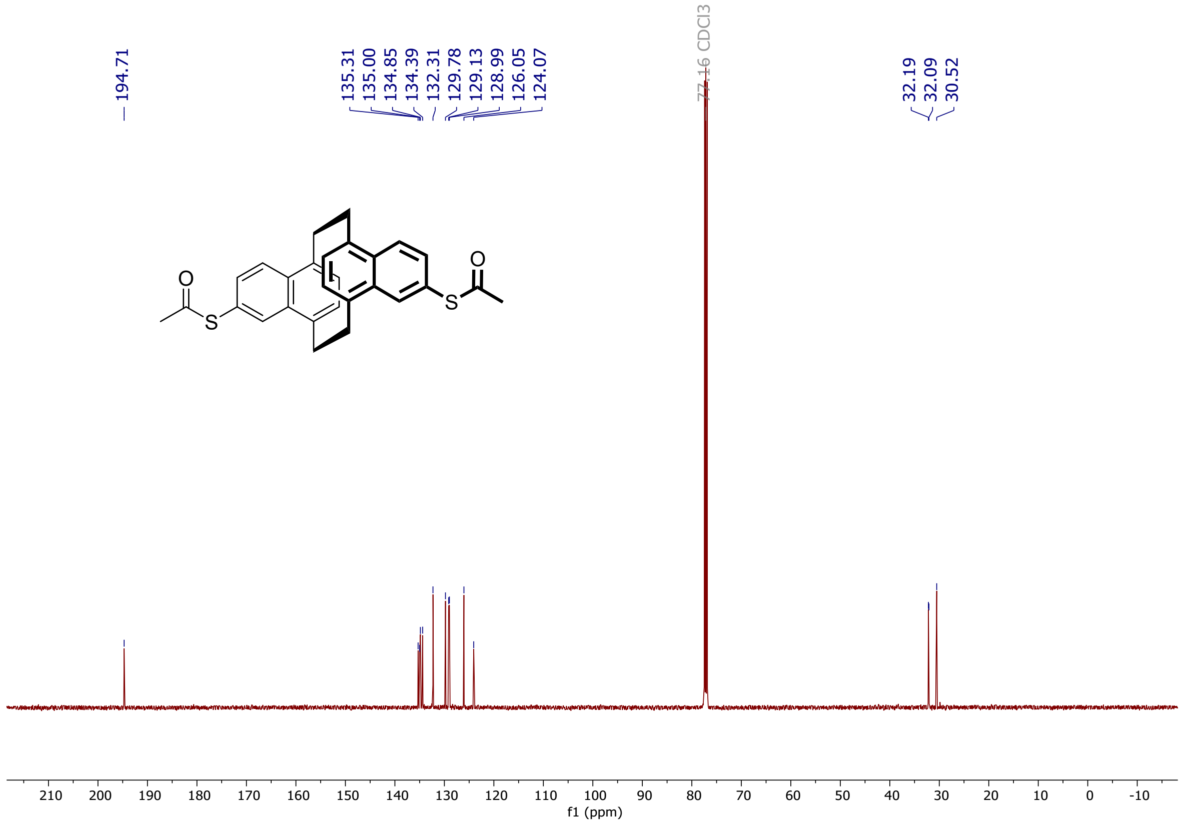

194.71
135.31
135.00
134.85
134.39
132.31
129.78
129.13
128.99
126.05
124.07
77.16 CDCl3
32.19
32.09
30.52
O
S
210 200 190 180 170 160 150 140 130 120 110 100 90 80 70 60 50 40 30 20 10 0 -10
f1 (ppm)

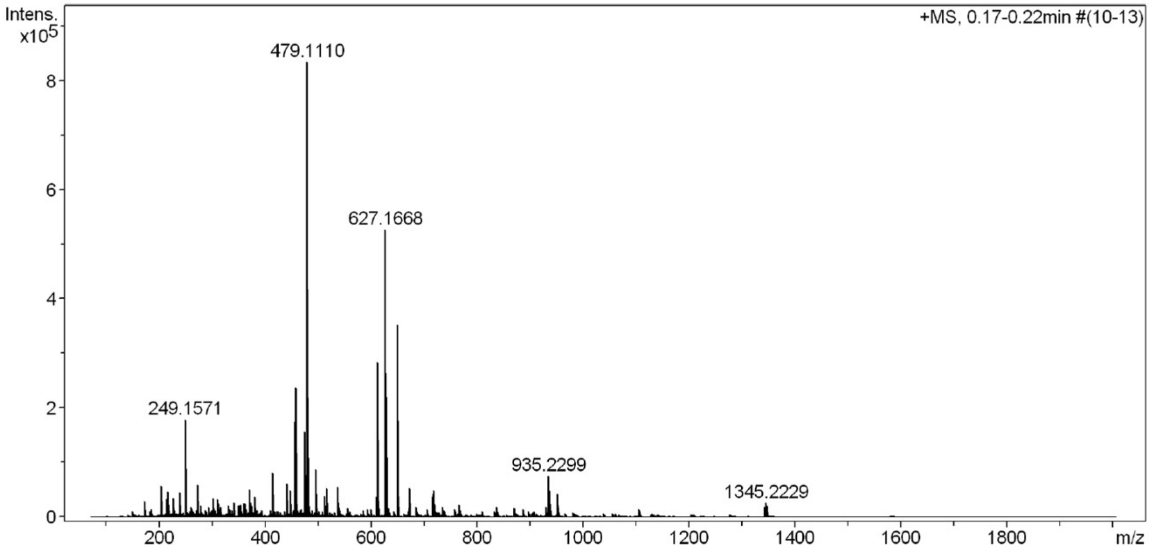

Intens.
x10^5
+MS, 0.17-0.22min #(10-13)
479.1110
627.1668
249.1571
935.2299
1345.2229
0 2 4 6 8
200 400 600 800 1000 1200 1400 1600 1800
m/z

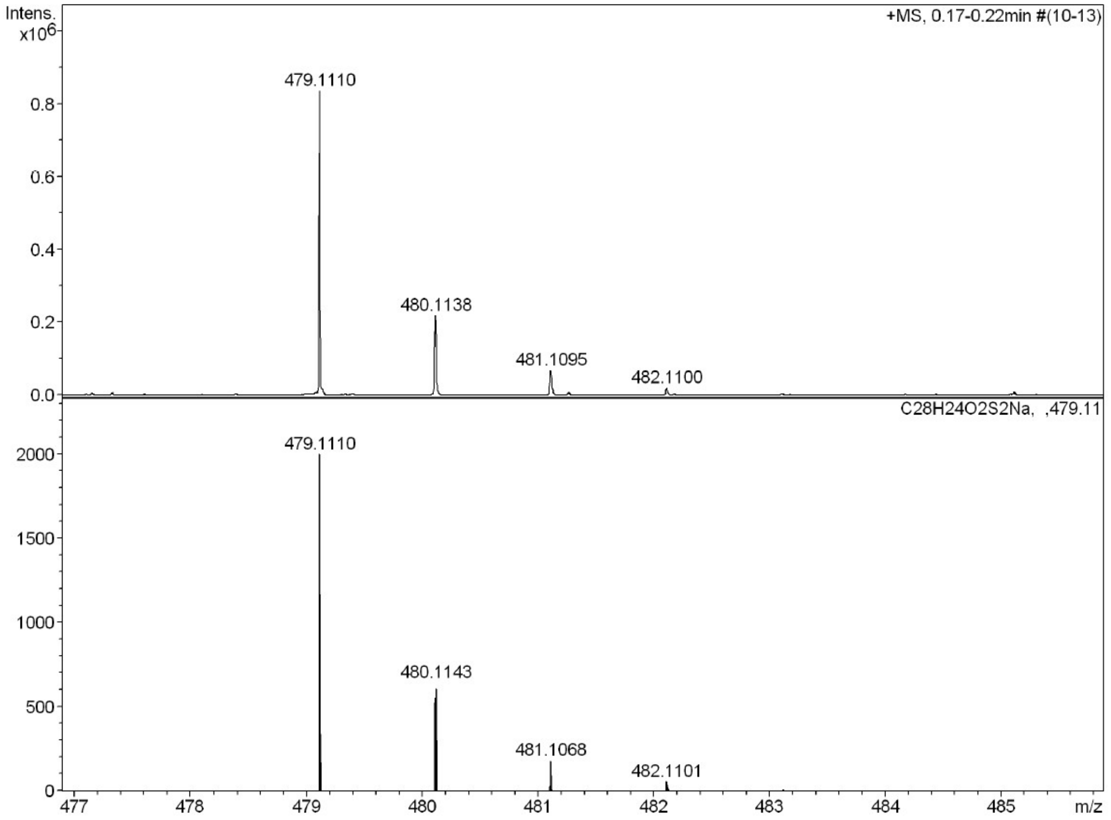

Measured m/z vs. theoretical m/z

Meas. m/z	#	Formula	Score	m/z	err [mDa]	err [ppm]	mSigma	rdb	e⁻ Conf	z
457.1286	1	C 28 H 25 O 2 S 2	100.00	457.1290	0.5	1.0	20.7	16.5	even	1+
474.1551	1	C 28 H 28 N O 2 S 2	100.00	474.1556	0.5	1.0	19.0	15.5	even	
479.1110	1	C 28 H 24 Na O 2 S 2	100.00	479.1110	0.0	0.0	37.3	16.5	even	
495.0842	1	C 28 H 24 K O 2 S 2	100.00	495.0849	0.7	1.5	17.4	16.5	even	

S2. MCBJ conductance measurements: reproducibility

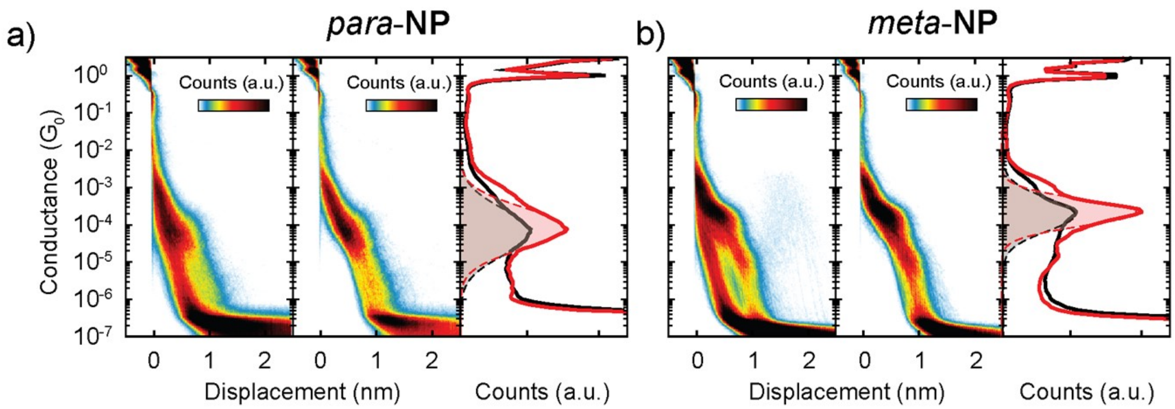

Figure S2: a) Conductance measurements of *para*-**NP**. Left and central panels show the 2D conductance vs. electrode displacement histograms, containing all the raw data and molecular data, respectively, obtained from MCBJ experiments. The raw data consists of 10,000 consecutive traces. The right panel shows the corresponding 1D histograms: raw data (black) and molecular data (red). Dashed lines are the Gaussian fits of the respective conductance peaks for raw and molecular data. **b)** Same as **a)** but for *meta*-**NP**.

Additional measurements have been performed on a different sample with the MCBJ technique to ensure reproducibility. Measurements were carried out in a similar fashion as in Fig. 3a and 3b, albeit using a different concentration of the dropcasted solution of the *para*-**NP** compound in DCM on the MCBJ sample. We used a 50 µM solution of *para*-**NP** instead of 5 µM, while the *meta*-**NP** concentration remained at 5 µM. As can be seen in Table S1, the obtained conductances and stretching lengths are close to the values reported in Table 1 of the main text. In the left panel of Fig. S2b a faint blue region is visible for displacement values longer than 1 nm and G values between 10^{-5} and $10^{-3}G_0$, whose origin is unclear. Due the unclear nature and its low yield (less than 1% of total data), these outliers are not shown in the molecular data that we extracted using the clustering technique.

Table S1: Most probable conductance (G_m) and apparent stretching length (L_s), considering all the molecular traces for all compounds in additional data sets (middle panels of Fig. S2a and S2b). Uncertainties, obtained from the standard deviations, are indicated for each value.

**MCBJ**			
para-**NP**		*meta*-**NP**	
G_m **(**G_0**)**	L_s **(nm)**	G_m **(**G_0**)**	L_s **(nm)**
$(7.9 \pm 3.3) \times 10^{-5}$	0.8 ± 0.1	$(1.9 \pm 0.6) \times 10^{-4}$	0.6 ± 0.1

S3. STM conductance measurements

Molecular compounds were deposited onto Au(111) samples using the drop casting technique. Au samples were annealed at approximately 900 K for 1-2 minutes, allowed to cool down to room temperature and then introduced into a 1 mM dichloromethane (DCM) solution of the corresponding molecule. After 20 minutes samples were dried off with nitrogen gas to eliminate possible molecular clusters on the surface. Mechanically cut Au wires (0.25 mm diameter, 99.99% purity, Goodfellow) were used as STM tips. A bias voltage was applied to the sample, using $V_{bias} = 100\ mV$. The tunnelling current was amplified using a double-stage, home-made, linear current-voltage (*IV*) converter with an overall gain of $2.5 \times 10^{10}\ V/A$ ($5 \times 10^{8}\ V/A$ in the first stage, multiplied by a factor of 50 in the second one). The Au tip is welded to a homemade printed circuit board (PCB) tip holder and bolted under a piezoelectric tube. With this system we can move the STM tip over the substrate vertically and horizontally with a resolution of $10 - 20\ pm$. All electronic signals are generated and read using a field programmable gate array (FPGA) with a $\pm 10\ V$ range. In the case of the piezoelectric tube signals, the FPGA output signals are amplified by a factor of 14 using a high voltage amplifier.

Breaking traces were acquired by recording the current while retracting the tip of the tip-sample junction. 2,200 traces are obtained for each compound. They are aligned by setting them to zero displacement, when the monoatomic Au junction between the tip and the sample is broken at a conductance of $1G_0$. In this way, we ensure a normalized analysis of all traces. The same procedure was followed for the MCBJ measurements.

S4. Apparent stretching length

For both break-junction techniques the apparent stretching length (L_s) of the molecular plateaus was obtained in the same way by fitting a Gaussian distribution to each conductance peak and determining the length differences of every trace between the Au-Au monoatomic breakage point (G_0) and $G_m + \Delta G$, where G_m and ΔG are the most probable conductance value and the standard deviation of the Gaussian fitting curves, respectively. These end points are represented in Fig. S3a as crosses on top of the individual breaking traces. To take into account multiple junction configurations, Gaussian distributions were fitted to one-dimensional displacement histograms, constructed from the trace length (L_{trace}) of individual breaking traces. The most probable value, L_s, was then determined as the maximum of the distribution (as shown in Fig. S3b).

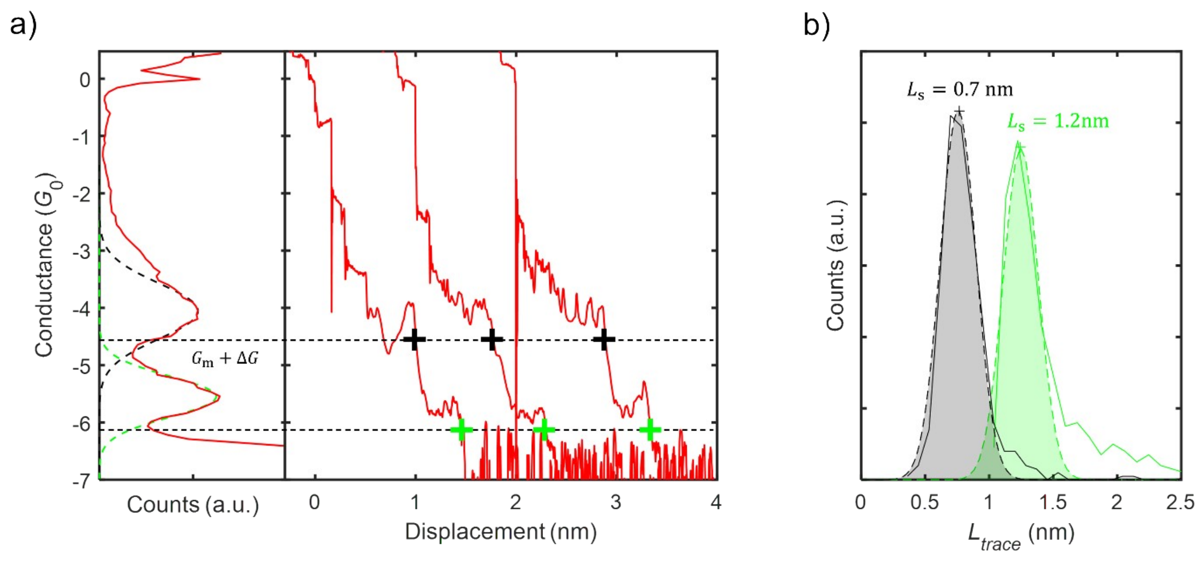

Figure S3: a) Left panel: 1D conductance histogram of the *para*-**NP** compound measured with the MCBJ technique. Dashed lines are the Gaussian fits to each G peak, black for the high-G plateau and green for the low-G plateau. Right panel: individual breaking traces. Grey dashed lines on top of both panels represent the $G_m + \Delta G$ value, where all the lengths are acquired. Black and green crosses on top of the breaking traces represent the apparent stretching length, L_{trace}, of high-G and low-G plateaus. Note that the breaking traces are offset horizontally for better visibility. **b)** Histograms compiling the lengths of traces (L_{trace}) for high-G and low-G plateaus from panel **a)** (solid lines). Peaks are fitted with Gaussian functions (dashed lines and filled areas), and crosses on top of the Gaussian fits represent the L_s value for each plateau.

S5. Clustering analysis

For both MCBJ and STM-BJ methods the same clustering analysis has been performed. The analysis applied was based on the k-means algorithm, provided by Matlab. To transform the G vs displacement traces into valid inputs for the algorithm, we created a vector with the elements of the 2D G vs displacement histogram and those of the 1D G histogram for each trace. The 2D G vs distance histogram of each trace was created using 40 bins on the G axis and 30 on the displacement axis, shaped as a 1,200-element vector. This vector is concatenated with a 100-element vector, containing the elements of the 1D G histogram of 100 bins for each trace. The resulting 1,300-element vector finally represents the algorithm input for each trace. G vs displacement traces are

limited in G and displacement to avoid current noise. For our region of interest the G limits are set to $[10^{-2}G_0, 10^{-6}G_0]$ and $[10^{-4}G_0, 10^{-6.5}G_0]$ for the 2D and 1D histogram, respectively, and $[0\ nm, 1.5\ nm]$ for the displacement of the 2D histogram.

With this technique traces with or without a molecular plateau were separated, and traces with different conductance plateaus could be divided into different clusters for each set of measurements. To obtain the cluster with a plateau at low G, limits for the input traces are changed to $[10^{-5}G_0, 10^{-6.2}G_0]$ for both the 2D and 1D histogram.

S6. High-conductance plateau

Individual breaking traces, containing only the high-conductance plateau, are displayed in Fig. S4 for both molecules. They stem from the molecular classes for both the *para*- and *meta*-**NP**, as presented in Fig. 3c and 3d. Both the MCBJ and STM methods give rise to similarly shaped single traces. Comparing the plateaus amongst the molecules, the plateaus in the single breaking traces of the *para*-**NP** contain more conductance variation than the plateaus of the *meta*-**NP**.

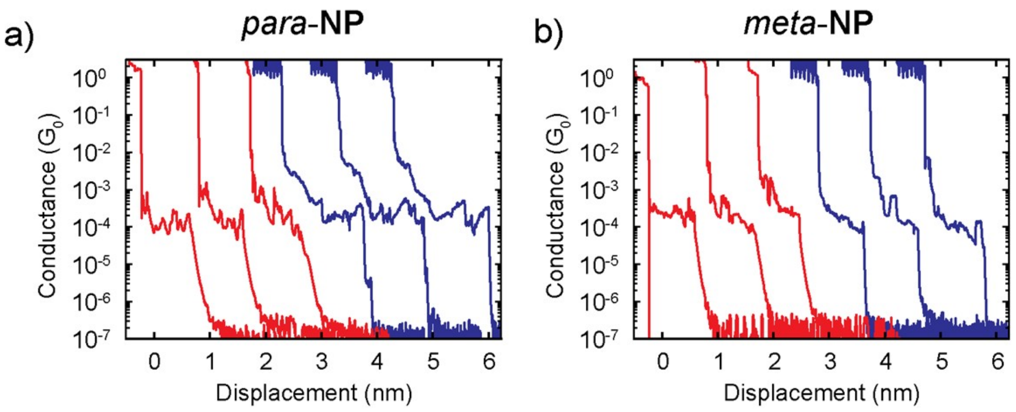

Figure S4: a) Examples of single breaking traces for *para*-**NP**, obtained from datasets as displayed in Fig. 3c and 3d, for both the MCBJ (red) and STM-BJ (blue) measurement methods. **b)** Same as **a)** but for *meta*-**NP**.

S7. Low-conductance plateau

One-dimensional conductance histograms of both compounds reveal a small conductance peak at lower values in addition to the main conductance maximum, indicating another possible stable configuration of the molecules inside the junctions. To learn about the origin, we applied the clustering technique of section S5 in order to create an ensemble that includes the traces with this low-conductance plateau. To achieve this, we changed the G parameters for the traces included in the clustering analysis to [$10^{-5}G_0$, $10^{-6.2}G_0$] for both histogram inputs. In Fig. S5 we show the 2D G vs. displacement and 1D G histograms containing all the traces with lower conductance plateaus for both compounds and methods. The most probable conductances (G_m) and apparent stretching lengths (L_s) of both plateaus are summarized in Table S2, representing the main conductance plateau as 'High *G*' and the low conductance plateau as 'Low *G*'. The classes were obtained by sub-clustering the molecular class in two classes, with molecular classes as shown in Fig. S2a and S2b for MCBJ and Fig. 3c and 3d for STM.

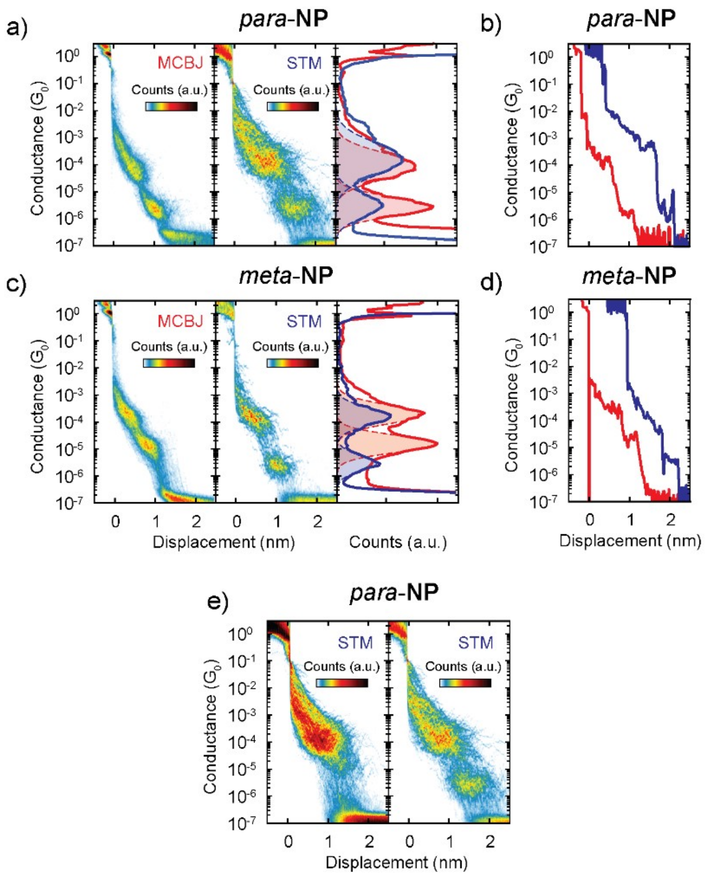

Figure S5: a) Conductance measurements of *para*-**NP**. Left and central panels show the 2D conductance vs. displacement histograms of the molecular traces with two conductance plateaus, obtained using the non-supervised clustering technique applied to MCBJ and STM results, respectively. The right panel shows the corresponding 1D histograms of MCBJ (red) and STM (blue) measurements, displaying two peaks in the conductance counts which correspond to the respective plateaus in the 2D histograms. Dashed lines are Gaussian fits to the respective conductance peaks for MCBJ and STM. **b)** Single trace examples of MCBJ and STM techniques with the red and blue lines obtained from the shown data sets for the *para*-**NP** in **a)**, respectively, which are offset for visibility. **c)** Same as **a)** but for *meta*-**NP**. **d)** Same as **b)** but for *meta*-**NP**. **e)** Example of the two sub-classes obtained from sub-clustering the STM molecular class in Fig. 3c. Note that the right panel is identical to the central panel shown in **a)**.

Table S2: Most probable conductance (G_m) and apparent stretching length (L_s) of the main high-conductance plateau ("High *G*") and low-conductance plateau ("Low *G*") for the molecular traces showing both conductance plateaus for *para*-**NP** and *meta*-**NP**, as measured with MCBJ and STM methods. Uncertainties, obtained from the standard deviation, are provided for each value.

	**MCBJ**				**STM**			
	para-**NP**		*meta*-**NP**		*para*-**NP**		*meta*-**NP**	
	G_m (G_0)	L_s **(nm)**	G_m (G_0)	L_s **(nm)**	G_m (G_0)	L_s **(nm)**	G_m (G_0)	L_s **(nm)**
High *G*	$(8.8 \pm 3.2) \times 10^{-[illegible]}$	0.8 ± 0.1	$(1.9 \pm 0.6) \times 10^{-[illegible]}$	0.6 ± 0.1	$(1.6 \pm 0.6) \times 10^{[illegible]}$	1.2 ± 0.2	$(1.6 \pm 0.6) \times 10^{-4}$	0.7 ± 0.1
Low *G*	$(2.7 \pm 1.8) \times 10^{-[illegible]}$	1.2 ± 0.2	$(1.6 \pm 0.9) \times 10^{-[illegible]}$	1.1 ± 0.2	$(3.2 \pm 0.9) \times 10^{[illegible]}$	1.7 ± 0.2	$(3.5 \pm 0.2) \times 10^{-6}$	1.2 ± 0.1

We find that 11%-15% of the molecular traces show a lower conductance plateau for the MCBJ and 20%-31% for the STM measurements. The lower conductance plateau is always accompanied by that preceding at a higher (main) conductance, as can be seen in the single trace examples of Fig. S5. Figure S5e shows an example of the two obtained classes from sub-clustering the molecular class, displayed in Fig. 3c and measured with the STM.

S8. STM-BJ Seebeck coefficient measurements

To perform Seebeck coefficient measurements a home-built STM was used, capable of measuring simultaneously the conductance (*G*) and the thermovoltage (V_{th}) of the formed molecular junctions. The tip was heated using a 1 kΩ surface resistor, creating a temperature difference (ΔT) between the tip and the sample, with the tip being at $T_h > T_{ambient}$ and the sample at $T_c = T_{ambient}$. This temperature difference not only generates a thermovoltage, V_{th}, in the molecular junction but also in the copper lead that connects the tip to the rest of the setup. Considering these factors the thermoelectric equation of the circuit can be expressed as

$$I = G(V_{bias} + V_{th}) = G(V_{bias} + S\Delta T - S_{lead}\Delta T), \quad (1)$$

where S and S_{lead} are the Seebeck coefficients of the molecular junction and the copper lead, respectively. Figure S6a shows a scheme of the equivalent circuit of the STM.

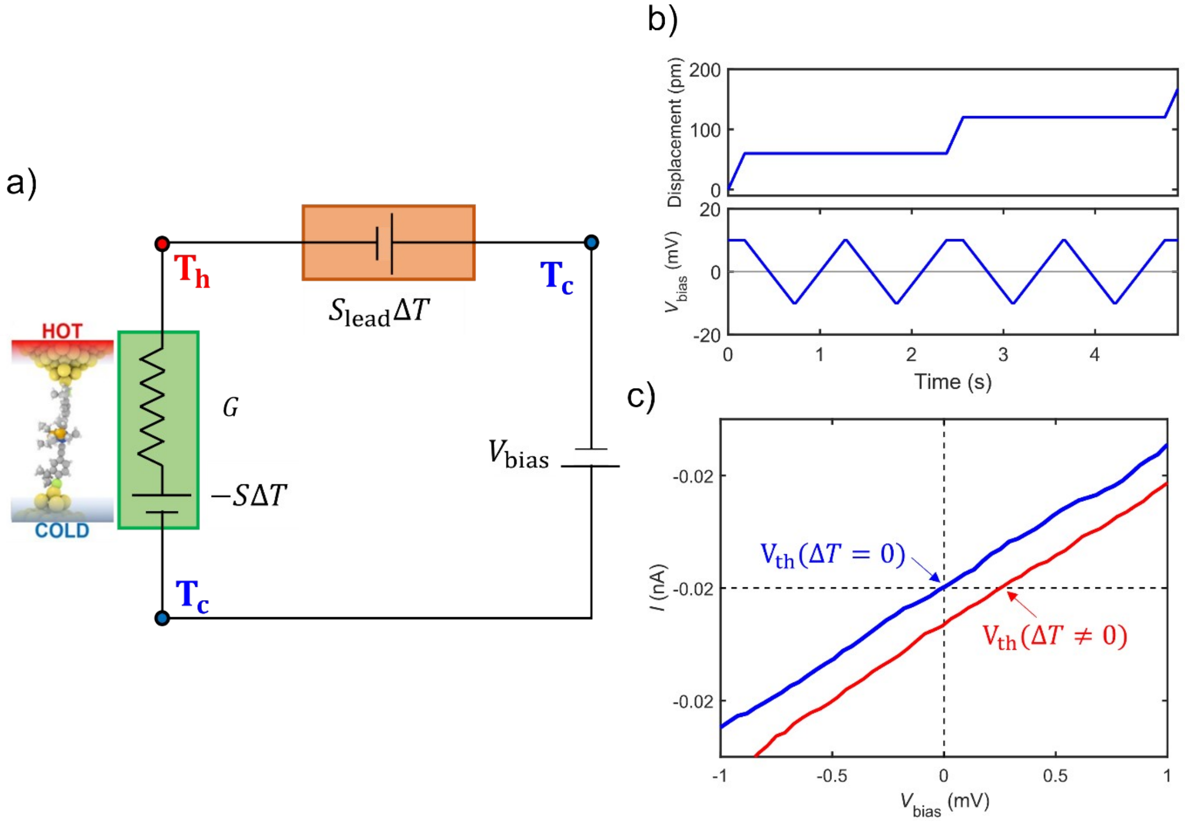

Figure S6: a) Scheme of the thermoelectric circuit of the STM, where V_{bias} is the bias voltage applied, S and S_{lead} are the Seebeck coefficients of the molecular junction and the copper lead, respectively, G is the conductance of the molecular junction, and ΔT is the temperature difference between the tip (at $T_h > T_c$) and the sample ($T_c = T_{ambient}$). **b)** (Top) Tip displacement z and (bottom) V_{bias} signals, respectively, during a thermovoltage measurement. While the molecular junction is formed, the tip displacement is momentarily stopped and V_{bias} is ramped between ±10 mV. **c)** Examples of current-voltage traces with temperature difference (red) and without temperature difference (blue), where V_{th} is obtained from the zero current crossing point and G from the slope of the trace.

In order to ensure a high mechanical stability of the junction, V_{bias} was limited to 10 mV, avoiding large voltage changes. While forming the molecular junctions, current-voltage curves for voltages between ±10 mV were acquired to perform the thermoelectric characterization. Examples of the tip displacement z and the bias voltage V_{bias}, applied in this case, are shown in Fig. S6b. The tip displacement is momentarily stopped during the junction formation, while the current-voltage

curves between ±10 mV are measured. Applying Eq. 1, V_{th} and G are simultaneously obtained from the zero-current crossing point and the slope of the current-voltage curves, respectively, and the Seebeck coefficient of the setup is then given by $S = V_{th}/\Delta T + S_{lead}$. Sets of V_{th} data were measured for different ΔT values and combined in order to obtain statistically robust results. Figure S6c shows an example of individual current-voltage traces with and without applied temperature difference, see the red and blue trace, respectively.

Figure S7 shows individual examples of simultaneous conductance and Seebeck coefficient measurements for the *para*-**NP** and *meta*-**NP** compounds. Top panels show the measured conductance vs. displacement trace (solid lines) and the conductance vs. displacement points (empty circles) where current-voltage curves were measured to quantify S. Bottom panels show the corresponding S values as empty circles and the S dependence on the displacement as a gently smoothed black solid line. An increase in S for decreasing G values is initially observed in most of the cases, while S tends to saturate for low G values and large electrode displacements lengths.

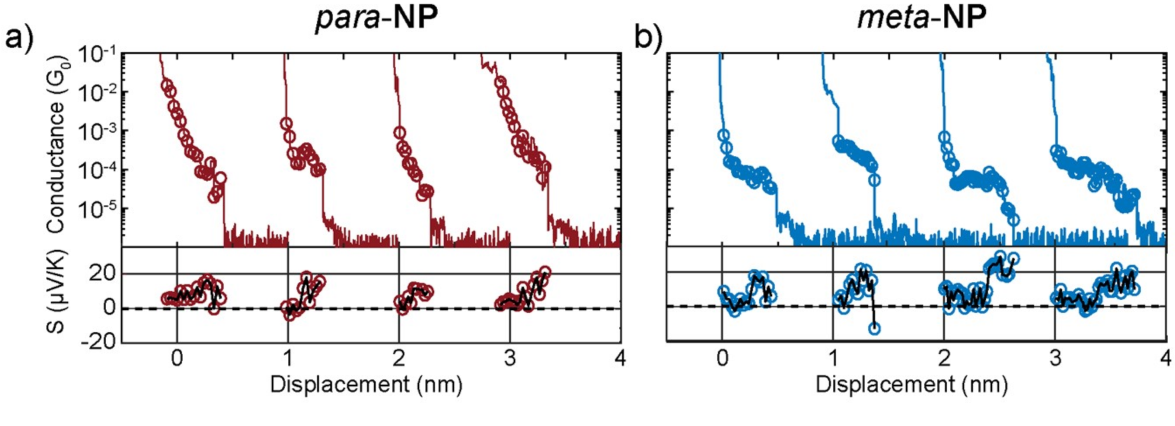

Figure S7: a,b) Examples of Seebeck coefficient (S) measurements for **a)** *para*-**NP** and **b)** *meta*-**NP** molecules. Upper panels display conductance vs. displacement traces and the points, where current-voltage traces are measured, as solid lines and empty circles, respectively. Lower panels show the S values extracted from each corresponding current-voltage measurement, indicated in the upper panel by an empty circle. Black lines represent a smoothed trace of S vs. displacement values.

II. Theoretical

S9. Four-site model

In addition to the theoretical analysis presented in Fig. 6 of the main text, we discuss here the four-site tight-binding model for the metal-molecule-metal junction with asymmetric couplings to left and right electrodes. This allows us to inspect the robustness of the model. Furthermore, we study the influence of variations in interdeck hopping d.

The four-site tight-binding model is depicted in Fig. S8a. As in Fig. 6, there are on-site energies $\varepsilon_i, i = 1,\ldots,4$, and hopping terms t and d. However, distinct couplings to left and right electrodes Γ_L and Γ_R are taken into account this time, which translate to the following Hamilton operator and linewidth broadening matrices:

$$\hat{H} = \begin{pmatrix} \varepsilon_1 & -t & 0 & 0 \\ -t & \varepsilon_2 & -d & 0 \\ 0 & -d & \varepsilon_3 & -t \\ 0 & 0 & -t & \varepsilon_4 \end{pmatrix}, \hat{\Gamma}_L = \begin{pmatrix} \Gamma_L & 0 & 0 & 0 \\ 0 & 0 & 0 & 0 \\ 0 & 0 & 0 & 0 \\ 0 & 0 & 0 & 0 \end{pmatrix}, \hat{\Gamma}_R = \begin{pmatrix} 0 & 0 & 0 & 0 \\ 0 & 0 & 0 & 0 \\ 0 & 0 & 0 & 0 \\ 0 & 0 & 0 & \Gamma_R \end{pmatrix} \quad (2).$$

We evaluate the transport properties using the Landauer-Büttiker approach[4] within the wide-band-limit approximation[5]. Since we do not consider any level shift inside the molecule from the coupling to the electrodes but only level broadenings, the self-energy matrices $\hat{\Sigma}_L = -i\hat{\Gamma}_L/2$ and $\hat{\Sigma}_R = -i\hat{\Gamma}_R/2$ are purely imaginary. The Green's function of the central device part reads

$$\hat{G}^r(E) = \left[E\hat{1} - \hat{H} - \hat{\Sigma}_L - \hat{\Sigma}_R\right]^{-1}, \quad (3)$$

with the identity matrix $\hat{1}$, and the transmission is given by $\tau(E) = \mathrm{Tr}\left[\hat{G}^a(E)\hat{\Gamma}_L\hat{G}^r(E)\hat{\Gamma}_R\right]$. Assuming a linear response regime with infinitesimally small differences in temperatures and electrochemical potentials between left and right electrodes, $\Delta T = T_L - T_R$ and $\Delta\mu = \mu_L - \mu_R$, results in the following expressions for the conductance G and thermopower S:[6–9]

$$G = G_0K_0, S = -\frac{K_1}{eTK_0} \, with \, K_n = \int dE\tau(E)\left(-\frac{\partial f(E)}{\partial E}\right)(E-\mu)^n. \quad (4)$$

Here, T is the average reservoir temperature, the electrochemical potential μ is assumed to be given by the Fermi energy E_F of the electrodes, $f(E) = \{exp[(E - \mu)/(k_B T) + 1]\}^{-1}$ is the Fermi function, $G_0 = 2e^2/h$ is the quantum of conductance, e is the (positive) elementary charge and h is the Planck constant. The explanations above obviously hold for the symmetric junction model of the main text, if $\Gamma = \Gamma_L = \Gamma_R$.

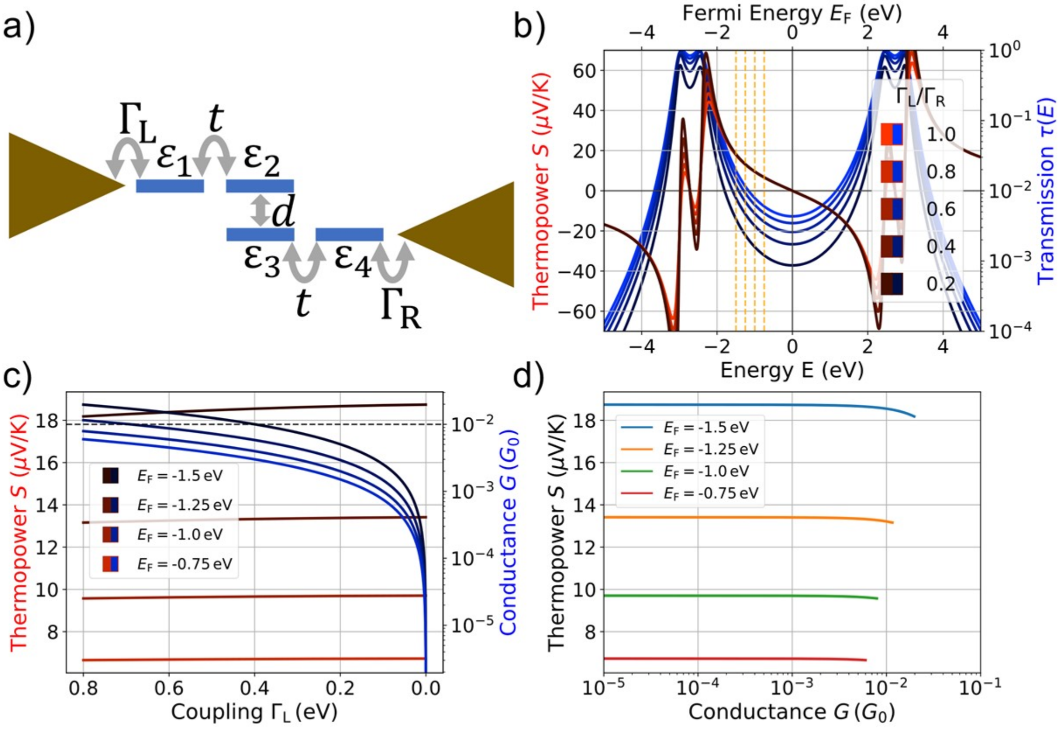

Figure S8: **a)** Four-site tight-binding model with on-site energies ε_i and $i = 1,\ldots,4$, intradeck hopping terms t and interdeck hopping d, allowing for asymmetric couplings to left and right electrodes through molecule-electrode couplings Γ_L and Γ_R. Model results are obtained with $\varepsilon_i = 0$ for all i, $t = 2.7\ eV$, and $d = 0.6\ eV$. For evaluations of the conductance G and thermopower S we assume a temperature of $T = 300\ K$. **b)** The transmission $\tau(E)$, plotted as a function of energy (lower x-axis), and the thermopower as a function of the respective Fermi energy (upper x-axis). Γ_R is kept fixed at 0.8 eV, and Γ_L is set according to the ratio indicated in the legend. **c)** S and G plotted against the coupling strength Γ_L for fixed $\Gamma_R = 0.8\ eV$. The behavior is depicted for the four different Fermi energies, which are indicated by vertical dashed orange lines in panel **b)**. The horizontal dashed line indicates a conductance of $10^{-2} G_0$. **d)** S plotted as a function of G, using the data from panel **c)**.

We can determine the energies of molecular transmission resonances from the eigenvalues λ_i of the Hamilton operator $\hat{H}$ as

$$\lambda_1 = 1/2\left(-\sqrt{(d^2+4t^2)}-d\right), \lambda_2 = 1/2\left(d-\sqrt{(d^2+4t^2)}\right), \quad (5)$$

$$\lambda_3 = 1/2\left(\sqrt{(d^2+4t^2)}-d\right), \lambda_4 = 1/2\left(\sqrt{(d^2+4t^2)}+d\right). \quad (6)$$

The parameter t represents the intradeck hopping within naphthalene units, while d parametrizes the interdeck hopping, which is typically smaller[10]. The HOMO-LUMO gap is then given by $\sqrt{d^2+4t^2}-d$, and the splitting of nearly degenerate HOMO and LUMO level pairs on the naphthalene units is determined by d. We use these relations to fit appropriate values for t and d from electronic structure calculations. For this purpose we calculate the energies of the frontier orbitals of an isolated *para*-**NP** structure with SH anchors attached, using density functional theory (DFT) as implemented in the TURBOMOLE program suite[11]. In the DFT calculations we employ the def-SV(P) Gaussian basis set[12] for all atoms and the PBE exchange-correlation functional[13]. Afterwards the energies of the frontier orbitals are corrected using a G_0W_0 calculation[14]. Since the HOMO levels for the equilibrium geometry are nearly degenerate whereas the LUMO levels exhibit a larger splitting, we fix the sulfur atoms and stretch the molecule along the sulfur-sulfur axis by 2 Å. Subsequently, a constrained geometry optimization is performed. In this way the close lying energies of the HOMO-1 and HOMO states are separated, and fitting yields the parameters $t = 2.7\ eV$ and $d = 0.6\ eV$.

Let us now analyze how the asymmetrically coupled four-site tight-binding model explains the experimentally observed behavior of decreased conductance and simultaneously growing thermopower during the stretching. As before, we study first a decrease in the coupling strength Γ_L or Γ_R due to the stretching, which can be justified by a reduced electronic overlap of molecule

and electrode[15–19]. In contrast to the analysis in Fig. 6 of the main text, we will thus keep one coupling fixed and lower the other coupling to simulate an asymmetric situation.

The results for the calculated transmission and thermopower with decreasing Γ_L, while $\Gamma_R = 0.8\ eV$ is kept constant, are depicted in Fig. S8b. Let us emphasize that the energy dependence in the expression for the thermopower S is integrated out. Therefore, we plot the thermopower in Figs. 6b and S8b against the corresponding Fermi energy. Variations of E_F are expected to be meaningful within the HOMO-LUMO gap, since there the trapped molecule will remain charge neutral. The broadenings of the molecular transmission resonances from HOMO-1 to LUMO+1 become smaller with decreasing Γ_L. This behavior is similar to the symmetric model. Different from the symmetric case, however, the maximum values of the transmission resonances are gradually reduced, deviating from the perfect transmission of 1. Inside the HOMO-LUMO gap region the transmission decreases with smaller Γ_L. Overall, the transmission is symmetric with respect to the middle of the HOMO-LUMO gap, i.e. electron-hole symmetric. The behavior of the thermopower S is comparable to the symmetric model. Absolute values of S near the transmission resonances are smaller, however, and the variation in S with changed Γ_L is reduced compared to the symmetric model. This is particularly evident from the analysis of S and G in Fig. S8c and S8d at the indicated Fermi energies. For $E_F = -0.75\ eV$, $E_F = -1.0\ eV$ and $E_F = -1.25\ eV$ almost no significant changes in the thermopower occur. The maximum relative change in the thermopower happens for $E_F = -1.5\ eV$, where the thermopower increases from $18.2\ \mu V/K$ to $18.7\ \mu V/K$ when Γ_L is reduced from $0.8\ eV$ to $0\ eV$. As for the symmetric model, the behavior of S versus G in Fig. S8d shows primarily a variation, when G decreases from high values at around $10^{-2} G_0$, while for lower values of G a saturation quickly sets in. Experimental trends of a reduced S for increased G are correctly reproduced by a growing left (or analogously right) molecule-electrode electronic

coupling. Due to the smaller range of variation in S, however, the agreement with the experimental data for the chosen parameters is somewhat less satisfactory on a quantitative level than for the symmetric model.

Finally, we examine the impact of changes in the interdeck hopping d on quantum transport properties. We choose a symmetric coupling $\Gamma = \Gamma_L = \Gamma_R$, so that the following explanations correspond to the scheme shown in Fig. 6a. An overview of the behavior of the thermopower S and the transmission $\tau(E)$ is given in Fig. S9a. A decrease in the parameter d results in a reduction of the splitting between the HOMO-1 and HOMO as well as LUMO and LUMO+1 energy levels, as evidenced by the eigenvalues λ_i (see Eqs. 5 and 6). Since the distance between the HOMO and LUMO states is mainly controlled by the large parameter t, which is held constant, a decrease in d results in a slight opening of the energy gap and a merging of the frontier orbitals into a single resonance. Overall, the transmission decreases at the indicated Fermi energies for smaller values of d. The thermopower decreases also. This can be seen in Fig. S9b as well as in Fig. S9c. However, the variation of the thermopower is not particularly sensitive to changes in d in the studied parameter range. Importantly, the qualitative trend is opposite to the experimental behavior (see Fig. 5), indicating that a change in d does not explain the global behavior of an increasing S for a reduced G. It should be noted that the linewidth broadening $\Gamma = 0.75\ eV$, assumed for the plots in Fig. S9, is comparatively high. For lower values of Γ, however, no qualitatively different behavior is found. Instead the relative changes in the thermopower S become even smaller, while the transmission continues to decrease.

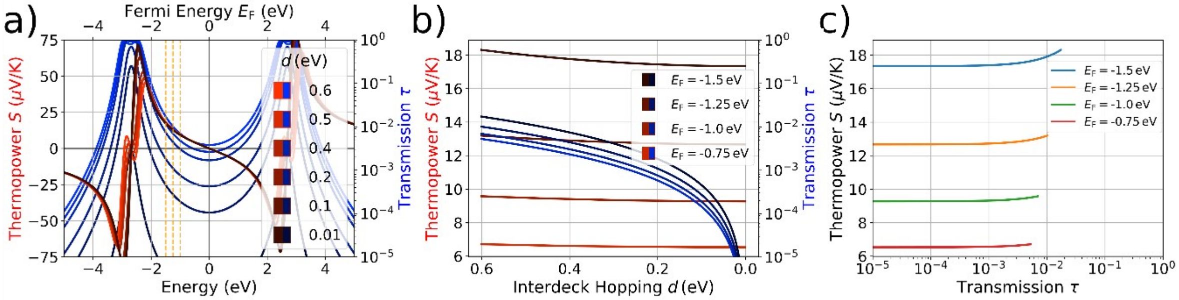

Figure S9: Four-site tight-binding model with on-site energies ε_i and $i = 1,\ldots,4$, intradeck hopping terms t and interdeck hopping d, and a symmetric coupling Γ to left and right electrodes. Model results are obtained for $\varepsilon_i = 0$ for all i, $t = 2.7\ eV$, $\Gamma = 0.75\ eV$ and a varying interdeck hopping d. For evaluations of the conductance G and thermopower S we assume a temperature of $T = 300\ K$. **a)** Transmission $\tau(E)$ plotted as a function of energy (lower x-axis) and thermopower as a function of the respective Fermi energy (upper x-axis) for different parameters d, indicated in the legend. **b)** S and G plotted against interdeck hopping d. The behavior is depicted for the four different Fermi energies, which are marked by vertical dashed orange lines in panel **a)**. **c)** S plotted as a function of G, using the data from panel **b)**.

S10. Single-level model

Simplifying even further to a symmetrically coupled single-level model, the behavior of S and G can be studied analytically. We model the broadened HOMO level at energy ε_0 by a Lorentzian transmission function[20]

$$\tau(E) = \frac{\Gamma^2}{(E - \varepsilon_0)^2 + \Gamma^2}. \qquad (7)$$

At low temperatures the conductance simplifies to $G = G_0 \tau(E_F)$, and the thermopower becomes $S = -\frac{\pi^2 k_B}{3e} \frac{\tau'(E_F)}{\tau(E_F)} k_B T$. Accordingly, the expression for the Lorentzian model reads

$$S = \frac{\pi^2 k_B}{3e} \frac{2(E_F - \varepsilon_0)}{(E_F - \varepsilon_0)^2 + \Gamma^2} k_B T. \qquad (8)$$

At constant Γ, an increased level offset $E_F - \varepsilon_0$ decreases $\tau(E_F)$ and thus $G = G_0 \tau(E_F)$, reducing S at the same time. This can clearly be seen from the relationship between S and $\tau(E_F)$ for a given Γ,

which reads $S = \frac{\pi^2 k_B}{3e} \frac{2}{|\Gamma|} \sqrt{\tau(E_F)(1 - \tau(E_F))} k_B T$ and decreases for decreasing $\tau(E_F) \leq 0.5$. Such a decrease of S with decreasing G contradicts the experimental observations in Fig. 5, and a level that is increasingly detuned from E_F while the molecule-electrode coupling stays constant can hence not explain the measurements.

Therefore, we study next, which effects changes in Γ have. For a constant level offset $E_F - \varepsilon_0$, a decreased Γ leads to a reduced G but increased S, resembling the experimental observations. The thermopower as a function of $\tau(E_F)$ at constant $E_F - \varepsilon_0$ reads $S = \frac{\pi^2 k_\mathrm{B} 2(1 - \tau(E_F))}{3e \quad E_F - \varepsilon_0} k_B T$. Thus, a linear relationship between S and $G = G_0 \tau(E_F)$ is expected in the off-resonant regime, if the molecule-electrode coupling remains symmetric and varies, while the level alignment is unaffected. For finite $E_F - \varepsilon_0$ and $\Gamma \to 0$, the transmission vanishes $\tau(E_F) \to 0$, and the thermopower reaches the saturation value $S \to \frac{k_\mathrm{B} \pi^2}{3e} \frac{2}{E_F - \varepsilon_0} k_\mathrm{B} T$.

The experimental trends can therefore be described with the help of the single-level model. However, we presented the four-site model in the main text as a minimalistic ansatz, where interdeck and intradeck electronic couplings better map the molecular structure of either *para*-**NP** or *meta*-**NP**. As discussed for the four-site model, for a quantitative explanation a complex simultaneous variation of both molecular orbital energies and level broadenings is required.

References

1 V. M. Cangelosi, A. C. Sather, L. N. Zakharov, O. B. Berryman and D. W. Johnson, *Inorg. Chem.*, 2007, **46**, 9278–9284.

2 S. Takahashi and N. Mori, *J. Chem. Soc., Perkin Trans. 1*, 1991, **1**, 2029–2033.

3 T. Yamamoto, A. Ishibashi, M. Koyanagi, H. Ihara, N. Eichenauer and M. Suginome, *Bull. Chem. Soc. Jpn.*, 2017, **90**, 604–606.

4 J. C. Cuevas and E. Scheer, *Molecular Electronics: An Introduction to Theory and Experiment,* 2nd Edition, World Scientific, 2017.

5 C. J. O. Verzijl, J. S. Seldenthuis and J. M. Thijssen, *J. Chem. Phys.*, 2013, **138**, 094102.

6 U. Sivan and Y. Imry, *Phys. Rev. B*, 1986, **33**, 551–559.

7 K. Esfarjani, M. Zebarjadi and Y. Kawazoe, *Phys. Rev. B*, 2006, **73**, 085406.

8 K. H. Müller, *J. Chem. Phys.*, 2008, **129**, 044708.

9 M. Bürkle, T. J. Hellmuth, F. Pauly and Y. Asai, Phys., *Rev. B*, 2015, **91**, 165419.

10 X. Li, A. Staykov and K. Yoshizawa, *Bull. Chem. Soc. Jpn.*, 2012, **85**, 181–188.

11 Y. J. Franzke, C. Holzer, J. H. Andersen, T. Begušić, F. Bruder, S. Coriani, F. Della Sala, E. Fabiano, D. A. Fedotov, S. Fürst, S. Gillhuber, R. Grotjahn, M. Kaupp, M. Kehry, M. Krstić, F. Mack, S. Majumdar, B. D. Nguyen, S. M. Parker, F. Pauly, A. Pausch, E. Perlt, G. S. Phun, A. Rajabi, D. Rappoport, B. Samal, T. Schrader, M. Sharma, E. Tapavicza, R. S. Treß, V. Voora, A. Wodyński, J. M. Yu, B. Zerulla, F. Furche, C. Hättig, M. Sierka, D. P. Tew and F. Weigend, *J. Chem. Theory Comput.*, 2023, **19**, 6859-6890.

12 A. Schäfer, H. Horn and R. Ahlrichs, *J. Chem. Phys.*, 1992, **97**, 2571–2577.

13 J. P. Perdew, K. Burke and M. Ernzerhof, *Phys. Rev. Lett.*, 1996, **77**, 3865–3868.

14 M. J. van Setten, F. Weigend and F. Evers, *J. Chem. Theory Comput.*, 2013, **9**, 232–246.

15 S. Park, J. Jang and H. J. Yoon, *J. Phys. Chem. C*, 2021, **125**, 20035–20047.

16 A. Torres, R. B. Pontes, A. J. R. da Silva and A. Fazzio, *Phys. Chem. Chem. Phys.*, 2015, **17**, 5386–5392.

17 S. Kobayashi, S. Kaneko, S. Fujii, T. Nishino, K. Tsukagoshi and M. Kiguchi, *Phys. Chem. Chem. Phys.*, 2019, **21**, 16910–16913.

18 S. Fujii, E. Montes, H. Cho, Y. Yue, M. Koike, T. Nishino, H. Vázquez and M. Kiguchi, *Adv. Electron. Mater.*, 2022, **8**, 2200700.

19 J. Vacek, J. V. Chocholoušová, I. G. Stará, I. Starý and Y. Dubi, *Nanoscale*, 2015, **7**, 8793–8802.

20 M. A. Popp, A. Erpenbeck and H. B. Weber, *Sci. Rep.,* 2021*,* **11***, 2031.*
